# Effectiveness and profitability of preventive veterinary interventions in controlling infectious diseases of ruminant livestock in sub-Saharan Africa: a scoping review

**DOI:** 10.1186/s12917-022-03428-9

**Published:** 2022-09-02

**Authors:** Francis Sena Nuvey, Jalil Arkoazi, Jan Hattendorf, Gloria Ivy Mensah, Kennedy Kwasi Addo, Günther Fink, Jakob Zinsstag, Bassirou Bonfoh

**Affiliations:** 1 Swiss Tropical and Public Health Institute, Kreuzstrasse 2, 4123 Allschwil, Switzerland; 2 Faculty of Medicine, University of Basel, Klingelbergstrasse 61, 4056 Basel, Switzerland; 3 Faculty of Science, University of Basel, Klingelbergstrasse 50, 4056 Basel, Switzerland; 4 Department of Bacteriology, Noguchi Memorial Institute for Medical Research, University of Ghana, P.O. Box LG 581, Accra, Ghana; 5 Centre Suisse de Recherches Scientifiques en Côte d’Ivoire, Abidjan, BP 1303 Côte d’Ivoire

**Keywords:** Effectiveness, Profitability, Preventive interventions, Ruminant livestock, Infectious disease, Vaccination, One health

## Abstract

Agriculture in general, and livestock production in particular, serve as a livelihood source for many people in sub-Saharan Africa (SSA). In many settings, lack of control of infectious diseases hampers livestock productivity, undermining the livelihood of rural populations. This scoping review sought to identify veterinary interventions previously evaluated as well as their relative effectiveness in controlling infectious livestock diseases. To be included, papers had to be written in English, German or French, and had to describe the effectiveness and/or profitability of preventive veterinary intervention(s) against anthrax, blackleg, bovine tuberculosis, brucellosis, contagious bovine pleuropneumonia, contagious caprine pleuropneumonia, foot-and-mouth disease, goat pox, lumpy skin disease, pasteurellosis, peste des petits ruminants, and/or sheep pox in any SSA country. Of the 2748 publications initially screened, 84 met our inclusion criteria and were analyzed. Most of the studies (*n* = 73, 87%) evaluated the effectiveness and/or profitability of vaccination, applied exclusively, applied jointly with, or compared to strategies like deworming, antimicrobial treatment, surveillance, feed supplementation, culling and dipping in reducing morbidity and/or mortality to livestock diseases. The effectiveness and/or profitability of antimicrobial treatment (*n* = 5), test and slaughter (*n* = 5), and use of lay animal health workers (*n* = 1) applied exclusively, were evaluated in the other studies. Vaccination was largely found to be both effective and with positive return on investment. Ineffective vaccination was mainly due to loss of vaccine potency under unfavorable field conditions like adverse weather events, cold chain failure, and mismatch of circulating pathogen strain and the vaccines in use.

In summary, vaccination is the most effective and profitable means of controlling infectious livestock diseases in SSA. However, to achieve effective control of these diseases, its implementation must integrate pathogen surveillance, and optimal vaccine delivery tools, to overcome the reported field challenges.

**Supplementary Information:**

The online version contains supplementary material available at 10.1186/s12917-022-03428-9.

## Introduction

Agriculture accounted for 28 and 16% of the gross domestic product of low income and lower middle-income countries in 2020 respectively [1, 2]. In sub-Saharan Africa (SSA), agriculture serves as pivot of employment, providing jobs to more than half of the workforce; a majority of jobs in rural areas and up to 25% of the jobs in urban areas [3]. Even though SSA accounted for the highest proportion of people employed in agriculture globally, more than 50% of its population [4], the region’s productivity in agriculture remain the lowest globally [1]. This coupled with having the highest population growth rate, 2.5% per annum, predisposes the region to food insecurity [5]. Therefore, more needs to be done to increase efficiency in production in order to improve the prospects of growth in the agricultural sector.

Agricultural production in SSA is dominated by the crop sector, which accounts for more than two-thirds of the production, measured in constant US dollars, although the share varies across the region with the highest (90%) and lowest shares (53%) in West and Southern Africa respectively [3]. In spite of being dominated by crops at the aggregate level, livestock production remain key to the livelihoods of many people; serving as the main protein source in the diet, source of income, store of wealth against uncertainties and as companion animals [6–9]. The majority of farmers in SSA engage in extensive livestock production. Thus, seasonality, availability of grazing resources, livestock diseases, security and conflict issues, and veterinary services availability affect their productivity greatly [10, 11].

Animal diseases are a major constraint to the development of the livestock sector, costing nearly 9 billion USD per year; about 6% of the value of the livestock sub-sector in Africa [12]. The high incidence and persistence of diseases in livestock in the region have been driven by a combination of factors including climate change, poor regulation of livestock movements, low utilization of preventive measures against diseases, and under-performance of veterinary services [13–15]. Animal diseases cause high mortality rates among livestock, especially in developing countries; diseases account for 7% of deaths in adult cattle, 21% of deaths in calves, 15% of deaths in adult sheep and goats and 23% of deaths in lambs and kids. Consequently, farmers lose on average one animal, for every animal sold in the case of large ruminants like cattle, and one animal for every two animals sold for small ruminants like sheep and goats to diseases [16]. Recent advances in science have shown a strong interface between human, animal and the environmental ecosystems, in terms of interdependence between the ecosystems and its associated heightened risks of disease transmission [17, 18]. Therefore, the lack of effective control of infectious livestock diseases do not only threaten animal health, but also poses significant threat to food security and public health.

For the most part, the provision of quality veterinary services enables countries to detect and control animal diseases effectively, thereby contributing to increased productivity of the livestock sector. The veterinary system delivers both curative and preventive services. With high utilization of particularly preventive veterinary strategies, the disease burden would be greatly reduced [9]. However, the delivery of veterinary services have been ineffective in many SSA countries, due to a limited participation of public and private veterinary personnel as well as livestock producers [19]. The poor performance of veterinary services in many African countries is mainly due to inadequate investment in veterinary services since the drastic changes in veterinary service policies in the 1980s, with only a handful of countries benefiting from these shifts in policy [20]. The shift in veterinary service policy is attributed largely to pressure from global financial institutions for developing countries to implement structural adjustment programs that promoted economic recovery to address high indebtedness levels, leading to the privatization of some veterinary services and reduction in the human, financial and material resources [19, 21, 22]. In addition, veterinary drug supply is poorly regulated in the region and the sector is dominated by private non-professional actors with commercial interest [23]. Strong investments in the veterinary services from both public and private sectors would therefore be key to achieving effective veterinary service delivery to improve farmer productivity. However, veterinary services have been chronically under-resourced, with a relatively low share of agricultural and health security investments, especially in developing countries leading to uncontrollable epidemics and high losses [9].

The World Organization for Animal Health (WOAH) instituted the Performance of Veterinary Services (PVS) Pathway to assist countries comprehensively assess the strengths and weaknesses of their veterinary services, and provide opportunities for resolution. A recent review of PVS appraisal reports of the veterinary services in Africa conducted in 2019 identified limited human, financial and material resources that affect particularly the delivery of field veterinary services as major barriers to effective control of diseases in Africa [24]. In addition, the low utilization of preventive veterinary services by livestock farmers remain a major bottleneck to the effective control of diseases [25]. Consequently, livestock diseases are ineffectively managed leading to a high burden of preventable infectious diseases and loss of livestock assets with large health, economic and psychosocial implications for farmers and the public at large [26, 27]. This scoping review was conducted to identify existing evidence in the SSA region regarding preventive veterinary interventions’ effectiveness and profitability in the control of selected infectious diseases in ruminants.

## Materials and methods

The study adopted the five-stage scoping review process proposed by Arksey et al. [28], namely identification of research question, identification of studies, selection of the relevant studies, data extraction and presentation of results. We also took recent recommendations by Peters et al. [29] into account for each stage of the scoping review process.

### Research questions related to the aims of the review

Our research question was “*what evidence exists regarding the effectiveness and profitability of preventive veterinary interventions for controlling infectious diseases in ruminants in sub-Saharan Africa?*” Specifically, we sought to answer the following three questions:

i. What interventions are or have been deployed to prevent infectious diseases in ruminants?
ii. How effective are these interventions in reducing the burden of infectious diseases in ruminants?
iii. How economically beneficial are these interventions?

The PICO elements were as described as follows:

- Population: ruminant livestock that are reared in sub-Saharan Africa
- Intervention: any strategy that is implemented with the aim of preventing or reducing the occurrence of infectious diseases in livestock
- Comparison: the comparison for the intervention, it could be a control group, a before-and-after comparison, or a comparison of use and non-use of the intervention in livestock
- Outcome: any documented outcome that describes the efficacy, effectiveness and/or profitability of the intervention on ruminant health

Any study published before May 11, 2021, was considered for inclusion in the review. The articles had to be in English, German, or French, and describe the effectiveness and/or profitability of preventive veterinary intervention(s) to be included in the review. The articles were screened for eligibility at the title, abstract and full paper review stages.

### Eligibility criteria and definitions

We defined a “preventive veterinary intervention” as any implemented strategy aimed at preventing or reducing the occurrence (prevalence or incidence) of infectious diseases in ruminants. Ruminant was defined as livestock domesticated for milk and meat production and comprises cattle, sheep, goat, camel and buffalo. The infectious diseases of interest were anthrax, bovine tuberculosis (bTB), blackleg, brucellosis, foot-and-mouth disease (FMD), contagious bovine pleuropneumonia (CBPP), contagious caprine pleuropneumonia (CCPP), lumpy skin disease (LSD), pasteurellosis, sheep pox, goat pox, and peste des petits ruminants (PPR). These diseases were selected based on a report outlining them as key infectious diseases affecting ruminant livestock in the West African region [18], priority diseases targeted for control in Ghana [30] as well as results from a previous study in Ghana [25].

### Study identification

We developed the search term for the review based on our research questions. With the assistance of professional librarians (library service of the University of Basel), we conducted an initial limited search and after evaluation refined the search terms. We applied the MeSH terms for each of the keywords and included the synonyms to improve the sensitivity of the search. We also used truncation to capture all possible uses of the keywords. The search term for sub-Saharan Africa, was adapted from the ISSG search filter resource, where we identified and refined the filter for use in PubMed [31, 32]. After the search strategy was optimized for PubMed, we then translated it using the SR-accelerator tool [33] developed by Bond University to generate the equivalent search term for Scopus. The search for the African Journals Online database was refined thereafter as it was less optimized for title/abstract and MeSH searches (see Additional file 1 for the search terms used). The searches were conducted on PubMed, Scopus and African Journals Online in May and June 2021, and included all studies published before then. We also manually searched the reference lists from authors of the included studies.

### Study selection

Two reviewers, FSN and JA, independently screened titles, abstracts and full texts and selected studies based on a priori inclusion/exclusion criteria. Studies were included if: *i)* they were published in English, French or German, *ii)* they employed observational (cross-sectional, case-control, cohort), secondary data analysis, and/or experimental designs, and *iii)* the title or abstract referred to or described the effectiveness and/or profitability of an intervention or strategy that aim to prevent or reduce the occurrence of any of the selected infectious diseases in ruminants.

Before the screening, FSN created an endnote library for all the articles retrieved from each search. The distinct endnote libraries were then merged and de-duplication automatically done using the “import into duplicates library” feature. Then, a manual de-duplication was done by screening the merged database to identify duplicates that were missed during the automatic process. The screening was done systematically according to the author names. Groups were created in the Merged EndNote library namely: relevant, irrelevant, duplicates, and no abstract or full-text unavailable for article classification. Relevant articles are those that meet the inclusion criteria. Irrelevant articles were articles that did not meet the inclusion criteria. Duplicates comprise all articles with multiple records. Articles without abstracts and/or full texts may be either relevant or irrelevant, after a retrieval of the articles by the library for screening.

Following this, the merged endnote library file was shared for independent screening of the article title and abstracts. The two reviewers met for the first time to review the a priori inclusion and exclusion criteria. After 1 week of independent screening, the two reviewers met again to compare notes on difficulties and identified strategies to overcome them. Where there were disagreements in classification of articles, the two reviewers met to resolve them by referring together to the a priori inclusion and exclusion criteria.

After the initial screening and classification, we also searched the cited references in the relevant articles for titles that could be relevant, screened and included the articles that met the inclusion criteria for the data extraction and analysis.

### Data extraction

Data were extracted by FSN and was reviewed by JH. The information extracted for each included study were author(s) of study, year of publication, year of study, country of study, objective of the study, livestock species studied, study design employed, data collection methods, and the intervention(s) evaluated. Other information extracted were details of the outcome(s) of interest, measure of effect or profitability of the intervention(s) and study limitations and conclusions. The data were entered into Microsoft Access and exported to Microsoft Excel for analysis.

### Synthesis of results

Given the broad range of eligible study types and research questions, outcomes and effect measures varied among studies. Therefore, it was not possible to generate a single summary measure of effectiveness or profitability. For studies that did not provide protective rates of intervention, but presented raw data on prevalence or incidence stratified by intervention and control groups, these analyses were done using the formula below.

$$\mathrm{Protective}\ \mathrm{rate}=\frac{disease\ prevalence\ or\ in cidence\ in\ control\ group- disease\ prevalence\ or\ in cidence\ in\ in tervention\ group}{disease\ prevalence\ or\ in cidence\ in\ control\ group}\times 100$$

In addition, we used data on benefits of intervention and intervention costs provided by studies that assessed profitability without reporting benefit-cost ratios (BCR), to estimate the BCR of implementing the intervention using the formula below.

$$\mathrm{BCR}=\frac{Benefits\ of\ the\ intervention=\left( Costs\ saved+ New\ revenue\right)}{Cost\ of\ the\ intervention= New\ intervention\ cost}$$

The results were presented as average protective rates of each intervention and for specific infectious diseases with their respective ranges. We also present average benefit-cost ratios for interventions applied for specific infectious diseases where applicable.

## Results

### Articles retrieved in the review

**Fig. 1:**
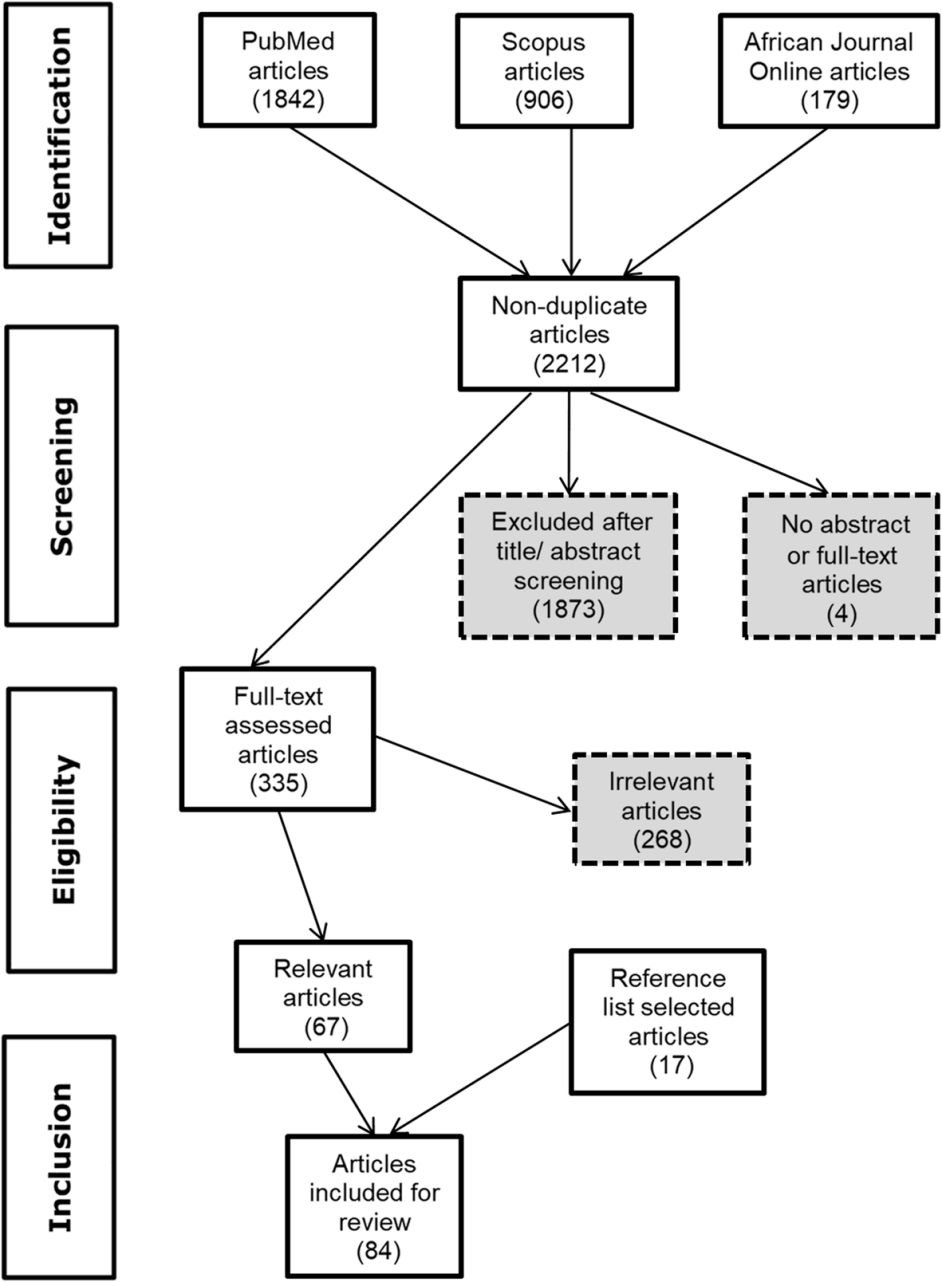
Steps followed during selection of studies for inclusion in the review

The literature search yielded 2927 hits; PubMed = 1842 hits, Scopus = 906 hits and African Journal Online (AJOL) = 179 hits. After removing duplicates in the merged database, 2212 articles were identified for title and abstract screening. Only four articles could not be retrieved for screening and were excluded. Many of the articles (85%, *n* = 1873) were excluded at the title and abstract screening stage because they either did not describe interventions against the infectious diseases of interest, were not implemented in sub-Saharan Africa, or employed study designs excluded in the protocol. After the full text review for eligibility (*n* = 335), 67 articles met the inclusion criteria. A further 17 articles were found from the reference lists of included articles. Thus, 84 articles were included for data extraction and analysis. Figure 1 shows the review process, following the Preferred Reporting Items for Systematic Reviews and Meta-Analyses–Extension for Scoping Reviews (PRISMA-ScR).

### Characteristics of the reviewed studies

**Fig. 2:**
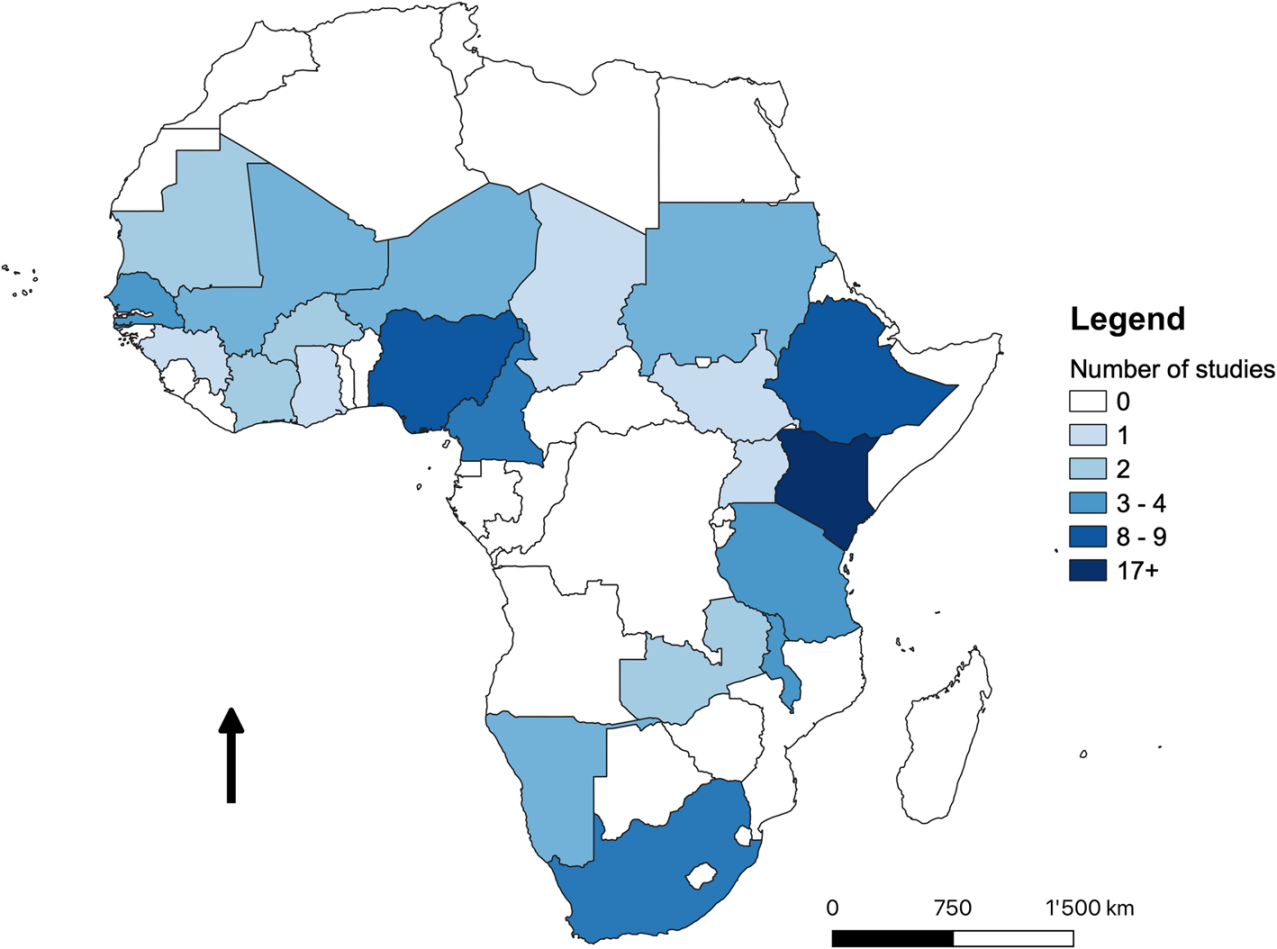
Geographical distribution of studies in the review

Out of the 84 publications reviewed, 40 (48%) were conducted in East Africa, 20 (24%) in West Africa, 14 (17%) in Southern Africa, 6 (7%) in Central Africa and 4 (5%) studies were done in multiple regions. The countries that dominated the published effectiveness and profitability of preventive veterinary interventions were Kenya (*n* = 24), Ethiopia (*n* = 17), Nigeria (*n* = 9), Cameroon (*n* = 8) and South Africa (*n* = 7) (Fig. 2). About half of the reviewed studies (*n* = 41) did not state the period during which they were conducted. For the studies (*n* = 43) that reported on the year of study, the earliest was done in 1954 and the latest in 2019. The studies were almost equally done in 20th and 21st centuries (before 2000, *n* = 20; after 2000, *n* = 23). In contrast, most of the studies (*n* = 60) were published after the year 2000.

The studies described interventions aimed at reducing morbidity and mortality in cattle (73%, *n* = 61), goats (14%, *n* = 12), mixed animal species (10%, *n* = 8), sheep (2%, *n* = 2) and buffalos (1%, *n* = 1). About 92% (*n* = 77) of studies evaluated interventions against only one infectious disease: CBPP (*n* = 28), FMD (*n* = 15), Bovine TB (*n* = 10), PPR (*n* = 9), LSD (*n* = 7), Blackleg (*n* = 2), Brucellosis (*n* = 2), CCPP (*n* = 2), and Pasteurellosis (*n* = 2). The remainder of the studies were on at least two of the above-mentioned infectious diseases in addition to anthrax and/or goat pox. Vaccination was the most frequently evaluated intervention; vaccination only (*n* = 63), vaccination applied in addition to or compared with other measures (*n* = 10), antimicrobial treatment (*n* = 5), test and slaughter (*n* = 5), and use of community animal health workers (*n* = 1).

Most of the studies (61%, *n* = 51) were experimental [under controlled conditions (*n* = 33), field trials (*n* = 17) and both under controlled conditions and field trial (*n* = 1)], 19% were cross-sectional studies (*n* = 16), and 10% were retrospective studies (*n* = 8). Some studies combined two or more designs; cross-sectional and retrospective data analysis (*n* = 5), cross-sectional and experiments (*n* = 1), cross-sectional, retrospective data analysis and longitudinal designs (*n* = 1). Two of the studies adopted a cohort design. Detailed characteristics of the studies are shown in Table S1 and Table 1.

**Table 1:** Summarized characteristics of studies reviewed

Variables	Description	Number of studies (references)
**Year study conducted**	Before year 2000	20 [34–53]
	Year 2000–2019	23 [54–76]
**Region of study**	West Africa	20 [34, 36, 44, 45, 47, 49, 50, 53, 57, 58, 68, 70, 73, 77–83]
	Central Africa	6 [51, 52, 59, 62, 84, 85]
	East Africa	40 [35, 38, 46, 54, 55, 60, 61, 63–67, 69, 71, 72, 74–76, 86–107]
	Southern Africa	14 [37, 39–43, 48, 108–114]
	Two or more regions	4 [56, 115–117]
**Objective of study**	Effectiveness of intervention	63 [34–45, 47, 50, 51, 53–66, 77–80, 84–105, 108–113, 115, 116]
	Cost-benefits of intervention	17 [67–76, 81–83, 106, 107, 114, 117]
	Effectiveness and cost-benefits	4 [46, 48, 49, 52]
**Study design**	Experimental study	51 [34, 36–41, 43–56, 59, 60, 62, 77, 79, 80, 84, 85, 87, 90–96, 99, 100, 102–105, 108–113, 115, 116]
	Cross-sectional study	16 [35, 42, 57, 58, 63, 65, 67, 70, 71, 73–76, 83, 89, 101]
	Secondary data analysis	8 [78, 81, 82, 88, 97, 107, 114, 117]
	Cohort study	2 [61, 72]
	Mixed (Two or more study designs)	7 [64, 66, 68, 69, 86, 98, 106]
**Animal species involved**	Cattle	61 [36, 39–42, 44–46, 48, 54–67, 69–71, 73–79, 81, 87–99, 101, 103, 104, 106–112, 114–117]
	Sheep	2 [49, 85]
	Goats	12 [34, 47, 50, 53, 72, 80, 82, 84, 100, 102, 105, 113]
	Buffalo	1 [43]
	Mixed (large and small ruminants)	4 [37, 51, 52, 83]
	Mixed (only small ruminants)	4 [35, 38, 68, 86]
**Disease studied**	Contagious bovine pleuropneumonia	28 [44, 45, 56–58, 69–71, 77–79, 89–99, 106, 108, 109, 115–117]
	Foot and mouth disease	15 [46, 59–63, 73, 74, 81, 101, 107, 110–113]
	Bovine tuberculosis	10 [39–43, 54, 55, 87, 88, 114]
	Pestes des petits ruminants	9 [50–53, 80, 82, 83, 85, 105]
	Lumpy skin disease	7 [64–66, 75, 76, 103, 104]
	Blackleg	2 [38, 67]
	Brucellosis	2 [36, 37]
	Contagious caprine pleuropneumonia	2 [72, 100]
	Pasteurellosis	2 [48, 49]
	Two or more infectious diseases	7 [34, 35, 47, 68, 84, 86, 102]

### Preventive veterinary interventions

The review revealed that the main preventive veterinary intervention was vaccination (*n* = 73, 87%) against the specified disease(s). The effectiveness and/or profitability of vaccination applied exclusively was evaluated in 63 of these studies. Nine studies evaluated effectiveness and/or profitability of vaccination plus: deworming (*n* = 4), antimicrobial treatment (*n* = 2), dipping (*n* = 1), and antimicrobial treatment and surveillance (*n* = 1). One study compared the effectiveness of feed supplementation versus vaccination applied jointly with deworming, while another study compared the profitability of vaccination, antimicrobial treatment and culling. The effectiveness and/or profitability of antimicrobial treatment (*n* = 5), test and slaughter (*n* = 5), and use of lay animal health workers (*n* = 1) applied exclusively, were also evaluated. Table 2 provides a summary of the interventions evaluated in the reviewed studies.

**Table 2:** Distribution of the interventions applied against the infectious diseases of interest

Intervention	Study design	Frequency	Study reference
**Vaccination only**	Anthrax	1	[86]
	Blackleg	3	[38, 67, 86]
	Bovine tuberculosis	6	[39–42, 54, 87]
	Brucellosis	1	[36]
	Contagious bovine pleuropneumonia	23	[44, 45, 57, 58, 69–71, 78, 79, 86, 89–99, 109, 116]
	Contagious caprine pleuropneumonia	3	[72, 86, 100]
	Foot and mouth disease	13	[46, 60–63, 74, 81, 101, 107, 110–113]
	Goat pox	2	[84, 102]
	Lumpy skin disease	7	[64–66, 75, 76, 103, 104]
	Pasteurellosis	2	[48, 86]
	Pestes des petits ruminants	9	[50, 51, 80, 82–84, 86, 102, 105]
**Vaccination applied jointly with other interventions**	Contagious bovine pleuropneumonia	3	[68, 115, 117]
	Anthrax	1	[34]
	Pasteurellosis	3	[34, 47, 49]
	Pestes des petits ruminants	6	[34, 47, 52, 53, 68, 85]
**Vaccination compared with other interventions**	Contagious bovine pleuropneumonia	1	[106]
**Antimicrobial treatment**	Contagious bovine pleuropneumonia	3	[56, 77, 108]
	Foot and mouth disease	2	[59, 73]
**Test and slaughter**	Bovine tuberculosis	4	[43, 55, 88, 114]
	Brucellosis	1	[37]
**Use of community animal health workers**	Anthrax	1	[35]
	Blackleg	1	
	Contagious caprine pleuropneumonia	1	

### Effectiveness of the interventions

**Fig. 3:**
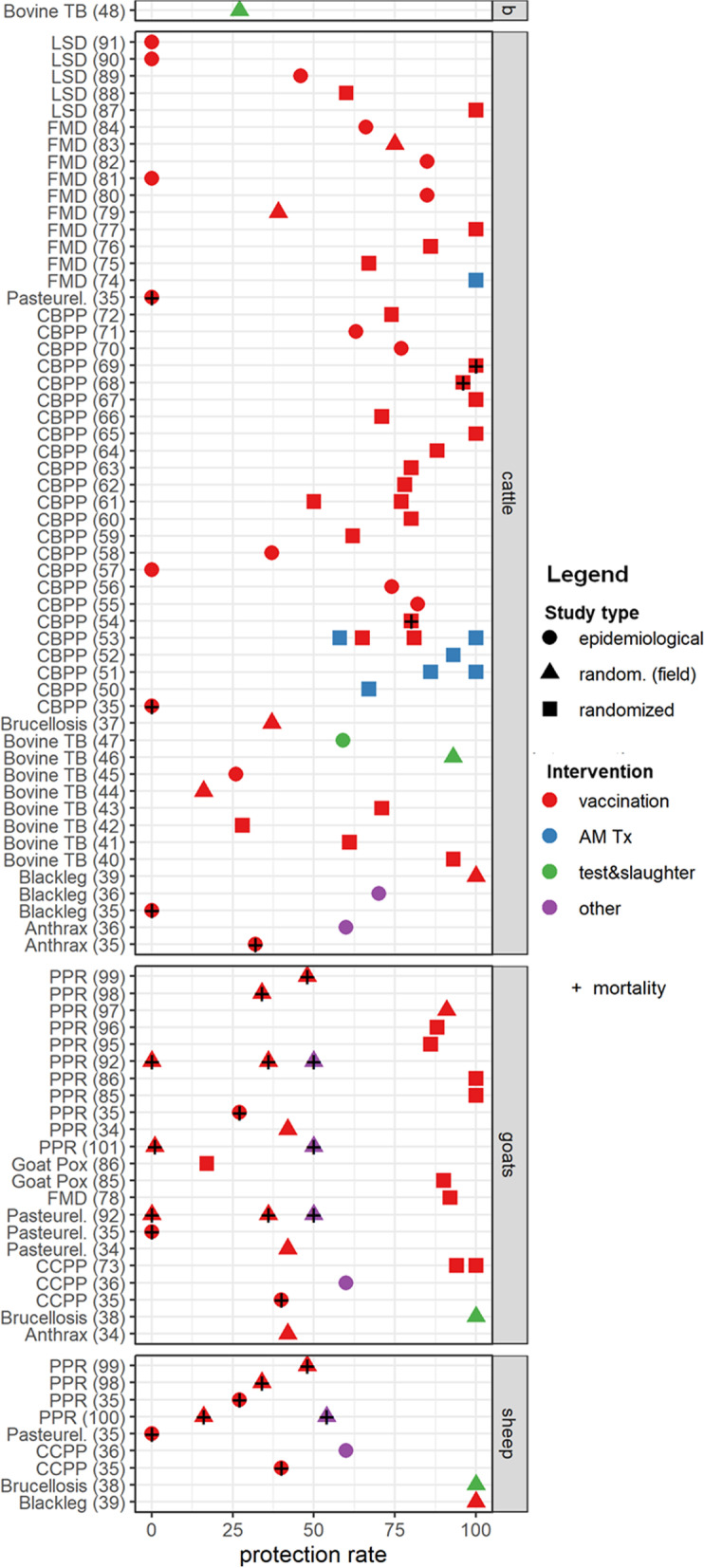
Effectiveness of preventive interventions against morbidity and mortality in ruminant livestock. The y-axis shows the specific diseases evaluated by species of livestock, with included study references in parenthesis. The x-axis shows the protection rates offered by the interventions against the specified diseases on a percentage scale. Interventions that did not offer protection against a disease in an included study have a 0% protection rate on the scale. Shapes are used to distinguish between study types while colors distinguish between the different preventive interventions evaluated in the included studies. “randomized” represents experimental studies implemented under controlled clinical conditions while “random. (field)” represents experimental studies implemented under natural field conditions. “epidemiological” denotes all other study types except experimental studies implemented in the included studies. “AM Tx” denotes antimicrobial treatment. “other” denotes the other interventions including deworming and dipping. The position of shapes on the percentage scale denote the protection rates of the interventions against morbidity to the specified diseases of interest in the included studies.”+” in a shape represents protection rate of the interventions against mortality to the specified disease. “b” denotes a study that evaluated test and slaughter strategy in buffalos

The effectiveness of the preventive interventions was evaluated in 75% (*n* = 63) of the reviewed studies while 5% (*n* = 4) evaluated both effectiveness and profitability. The effectiveness assessment was either for single interventions (*n* = 60), or a combination package of interventions (*n* = 7). To evaluate effectiveness, 43% (*n* = 36) of the studies computed morbidity or mortality rate differences or ratios between intervention and control groups. One-third of the studies (*n* = 31) reported protective rates of the implemented intervention(s) against morbidity and/or mortality in intervention and control groups. Figure 3 shows the effectiveness of interventions evaluated for each disease. We provide a summary of the effectiveness of the interventions implemented across the studies by each infectious disease below.

### Anthrax

Three studies evaluated the effect of preventive interventions on anthrax in cattle and goats. Two of the studies assessed mortality rate differences in vaccinated and unvaccinated cattle and goats, while the other study assessed the impact of community animal health workers’ (CAHWs) management of anthrax in rural Ethiopia.

The effectiveness of vaccination could only be assessed in one of the vaccination studies as the other compared mortality rates among goats receiving vaccines against three diseases (pasteurellosis, anthrax and PPR). Thus, only a joint effectiveness of the three vaccines could be evaluated. The overall effectiveness of the vaccines was 34% protection in goats less than 6 months old, and 50% protection in adult goats [34]. The effectiveness of vaccination in the other study was mixed; vaccination appeared effective during drought years (protection rate = 64%). However, during a normal year’s vaccination, it was not protective [86].

The evaluation of effectiveness of CAHWs deployment showed that, the effect of anthrax in pastoralists’ herds reduced by 60% following the activities of CAHWs in the communities [35].

### Brucellosis

Both studies on Brucellosis were field trials; one evaluated vaccination as an intervention while the other evaluated a test and slaughter approach. The outcomes assessed were different in the two studies. Both interventions were effective; vaccination offered a 37% protection rate against brucellosis-related abortions and still births in cattle [36] while test and slaughter was 100% protective against brucellosis infections in sheep and goats [37].

### Blackleg

Three studies evaluated effectiveness of preventive interventions against Blackleg in cattle and sheep. The studies adopted experimental, cross-sectional and retrospective study designs. The interventions evaluated were vaccination and CAHWs deployment. The outcomes of interest varied across the studies. The deployment of animal health workers was effective, reducing the effect of blackleg in pastoralists’ herds by 70% following the activities of CAHWs [35]. However, the effectiveness of vaccination was unclear. In an experimental study, the authors observed a protective rate of 100% against blackleg related deaths in cattle [38]. However, a retrospective review of data in another study found vaccination to be ineffective [86].

### Bovine tuberculosis

Eight out of the nine studies that evaluated effectiveness of interventions against Bovine tuberculosis (bTB) were done in cattle while the other was done in buffalos. The interventions mainly evaluated were vaccination (*n* = 6) and test and removal (*n* = 3). The effectiveness of vaccination was evaluated under controlled conditions in four out of the six studies. Both vaccination and the test and slaughter strategies were protective against bTB infection and/or deaths in all the studies, although the protection rates varied.

In the six studies that evaluated the protection rate of vaccination against bTB infection in cattle [39–42, 54, 87], an average protective rate of 63% (range: 28 to 93%) under controlled conditions in clinical trials and 21% (range: 16 to 26%) under natural field conditions was reported. The results for the test and removal strategy were not different either. In cattle, test and slaughter strategy provided an average protection rate of 76% (range: 59 to 93%) against bTB infection [55, 88]. However, in buffalos test and slaughter offered a protection rate was 27% [43].

### Contagious bovine pleuropneumonia

Twenty-three studies evaluated the effectiveness of interventions against contagious bovine pleuropneumonia (CBPP) morbidity and/or mortality in cattle. CBPP is the only disease for which interventions were evaluated in all the sub-Saharan African regions. The outcomes of interest in these studies varied but all interventions implemented were generally effective. To assess the extent of CBPP morbidity, most of the studies adopted the Hudson and Turner approach [118] in lesion scoring.

Three of the studies (experiments) evaluated the protective rate of antimicrobial treatment [danofloxacin [108], long acting oxytetracycline [77] and tulathromycin and gamithromycin [56]] against CBPP infection and infection spread among cattle under controlled conditions in clinical trials. Overall, the antimicrobials used were efficacious against CBPP morbidity; average protection rate was 82% (range: 67 to 93%).

A trial assessed both vaccination and treatment approaches against CBPP infection and deaths. The study reported an average protection rate of 65% against morbidity and 81% against mortality, for the 2 vaccine formulations tested. The authors observed that treatment with oxytetracycline protected infected animals against the extension of lesions in the lungs (protection rate = 58%) [115].

Even though the studies evaluating the effectiveness of vaccination alone [44, 45, 57, 58, 78, 79, 86, 89–99, 109, 116] reported mixed results, the evidence shows vaccination to be effective against both CBPP morbidity and mortality. All studies evaluating vaccination under controlled conditions (*n* = 13), were highly protective against CBPP infection and deaths: average protection rate against CBPP infection was 77% (range: 50 to 100%) and mortality was 92% (range: 77 to 100%). In the seven other studies that evaluated vaccination against CBPP, only five showed vaccination to be effective; average protective rate against CBPP infection was 67% (range: 37 to 82%). In the two cross-sectional studies where vaccination was ineffective, prevalence of infections and deaths from CBPP were higher in cattle with a history of vaccination.

### Contagious caprine pleuropneumonia

Three studies evaluated effectiveness of interventions (vaccination and community animal health workers deployment) against contagious caprine pleuropneumonia (CCPP) in goats and sheep. Both interventions were effective against CCPP infection in the studies. Protective efficacy of vaccination against morbidity and mortality in goats were 94 and 100% respectively in an experiment under controlled conditions [100]. A retrospectives study found a lower protective rate of vaccination (40%) against CCPP mortality in sheep and goats [86]. The study that evaluated CAHWs deployment found the effect of CCPP in pastoralists’ herds to reduce by 60% following the activities of CAHWs in the communities [35].

### Foot-and-mouth disease

Ten out of eleven studies assessed the effectiveness of vaccination against foot-and-mouth disease. The other intervention evaluated the efficacy of a novel topical anesthetic and antiseptic formulation (Tri-Solfen) against FMD lesions. Only one study assessed intervention effectiveness in goats; the rest were all in cattle.

The comparison of the efficacy of Tri-Solfen and antimicrobial treatment (parenteral oxytetracycline) against FMD lesion healing under controlled conditions in a trial showed a 100% protective rate of both treatments towards clinical recovery, but with a more rapid healing observed for the new formulation compared to the parenteral oxytetracycline group [59].

Vaccination was highly protective against FMD infection in all the studies done under controlled conditions in clinical trials (*n* = 4): average protection rates were 84% in cattle (range: 67 to 100%) and 92% in goats [110–113]. In the six studies evaluating effectiveness of vaccination against FMD infection in cattle under natural field conditions [46, 60–63, 101], only one was ineffective. Average protection rate across the studies was 70% (range: 39 to 85%). In the cohort study where vaccination was ineffective, incidence of FMD infection during an outbreak was highest in cattle with previous histories of vaccination against FMD; the risk of infection increased with an increase in the lifetime doses of FMD vaccines received by the cattle [61].

### Goat pox

Two experiments assessed the efficacy of vaccination against goat pox infection under controlled conditions. The protection rate of vaccination against goat pox infection differed widely in the two studies. While goats vaccinated against goat pox were fully protected in one study [102], the other study reported a protection rate of only 17% [84].

### Lumpy skin disease

Five studies evaluated the effect of vaccination against lumpy skin disease (LSD) infection in cattle. Vaccination was highly protective against LSD infection in the two studies [103, 104] done under controlled conditions; average protection rate was 80% (range: 60 to 100%). Only one of the other three studies done under natural field conditions found vaccination to be protective against LSD infection; protection rate was 46% [64]. The two other cross-sectional studies observed a higher prevalence of infections and deaths in vaccinated compared to the unvaccinated cattle [65, 66].

### Pasteurellosis

Five studies evaluated effectiveness of interventions against pasteurellosis morbidity and mortality in livestock under natural field conditions. Two of these studies assessed in addition the combined effects of multiple vaccines and deworming in a parallel group [34] or factorial design [47]. The net effect in both studies was that both treatments were effective in reducing mortality rates in goats, the effect even more profound when vaccination and deworming are combined.

In the three other studies, the effectiveness of vaccination was unclear. Due to differences in the outcomes of interest, a pooled estimate of protection rate could not be derived. One of these studies compared the efficacy of two vaccine formulations and found a modified vaccine to be about 15% more efficacious than the standard vaccine in preventing pasteurellosis infection in cattle [48]. In a retrospective study, vaccination was not protective against pasteurellosis related deaths in cattle in both normal and drought years, and in goats and sheep during drought years, but was protective (protection rate = 18%) in sheep and goats when vaccination was done in normal years [86]. In another experiment, Lesnoff et al. [49] showed vaccination alone was ineffective, but deworming alone or vaccination applied jointly with deworming improved productivity (reduced mortality and increased fecundity) in goats.

### Peste des petits ruminants (PPR)

Twelve studies evaluated the effectiveness of interventions against PPR morbidity in goats and sheep. Two of these studies described a combined effect of multiple vaccines and had been reported earlier [34, 47]. In all the other studies, the effects of either PPR vaccination, feed supplementation, deworming and/or pest control on PPR infection and deaths were evaluated.

Overall, vaccination has been shown to be effective in PPR control. Under controlled conditions in clinical trials (*n* = 4), vaccination provided an average protection of 94% against PPR infection (range: 86 to 100%) and 100% protection against PPR related deaths in goats [80, 84, 102, 105]. Under natural conditions, protection is slightly lower; protection rate against PPR infection was 91% [50] and against PPR related deaths, protection rate was 31% on average (range: 27 to 34%) in sheep and goats [51, 86].

The other three studies evaluated the effectiveness of vaccination in addition to other measures including dipping, deworming and feed supplementation. The application of vaccination jointly with deworming provided a protection rate of 48% against mortality in small ruminants [52]. However, providing feed supplement was more protective against mortality in sheep than vaccination and deworming applied jointly [85]. While, dipping was more effective against mortality in goats when applied alone, than when applied jointly with vaccination [53].

### Profitability of the interventions

**Fig. 4:**
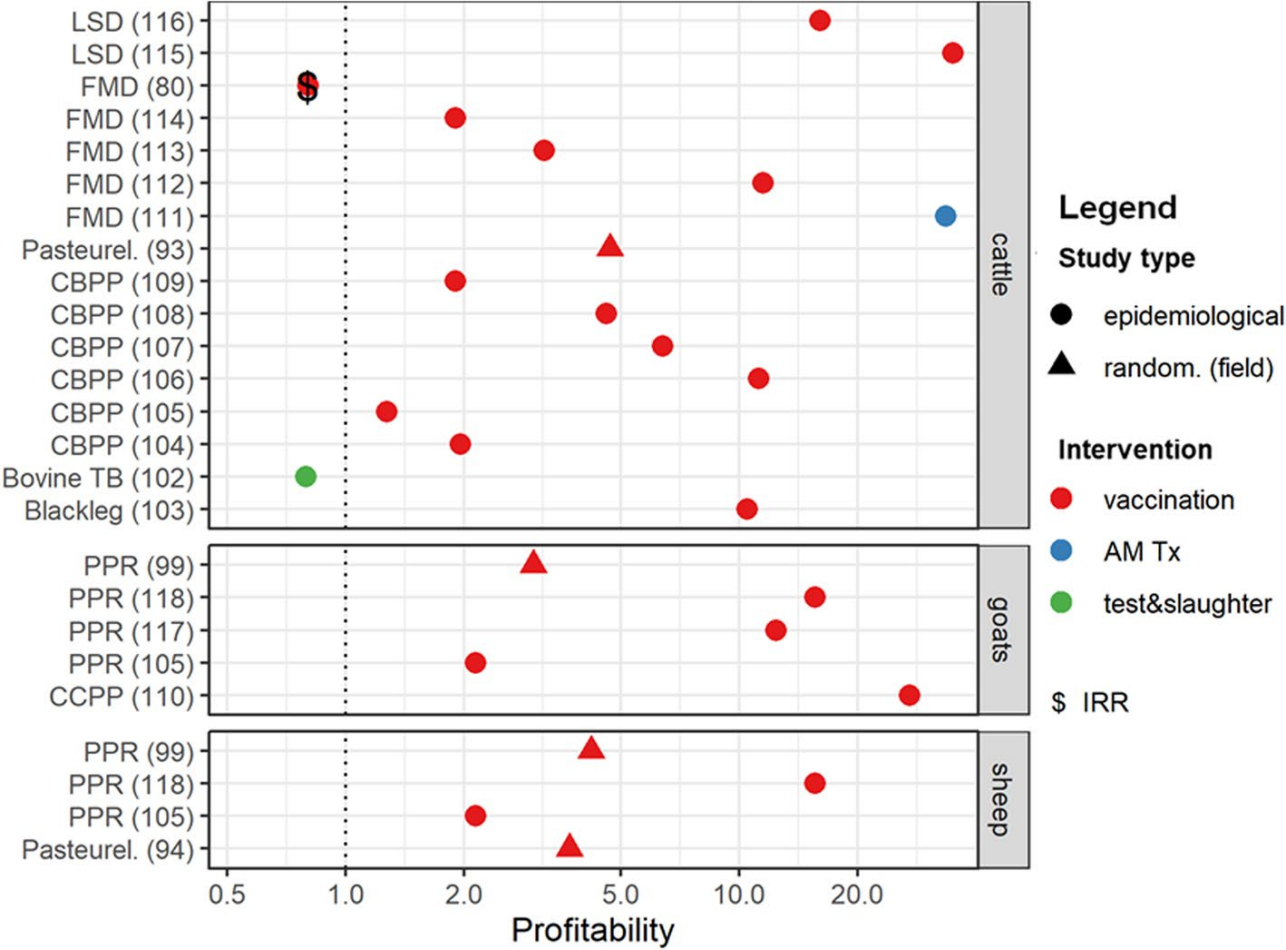
Profitability of preventive interventions in controlling infectious diseases in ruminant livestock. The y-axis shows the specific diseases evaluated by species of livestock, with included study references in parenthesis. The x-axis shows the profitability of the interventions in controlling the specified diseases on a benefit-cost ratio (BCR) scale. Shapes are used to distinguish between study types while colors distinguish between the different preventive interventions evaluated in the included studies. “random. (field)” represents studies that adopted field trials while “epidemiological” denotes all other study types except experimental studies. “AM Tx” denotes antimicrobial treatment. The position of shapes on the BCR scale denote the profitability of the interventions in controlling the specified diseases of interest in the included studies. “$” in shapes represents the internal rate of return of an intervention in controlling the specified disease for studies that did not present data for BCRs to be calculated

About 25% (*n* = 21) of the reviewed studies evaluated profitability of implemented interventions; four of these studies evaluated both effectiveness and profitability. The majority (*n* = 15) of the studies reported benefit cost-ratios (BCR), 3 studies reported marginal rate of return (MRR), 2 reported internal rate of return (IRR) and 1 reported the net return of implementing the intervention(s). The profitability analyses was done only for blackleg, bTB, CBPP, CCPP, FMD, LSD, pasteurellosis and PPR control strategies. Figure 4 shows the profitability of interventions for controlling the infectious diseases of interest. Overall, apart from strategies involving culling of infected animals, all other interventions evaluated provided positive returns on investment. We present below a summary of the profitability for interventions for controlling each of the diseases.

### Bovine tuberculosis

The profitability of a test and slaughter strategy in controlling bTB morbidity and mortality in both cattle and humans was assessed in one study. The benefit cost ratio was 0.79, thus the costs of control always exceed the benefits if purely monetary estimates were considered [114].

### Blackleg

The control of blackleg in cattle was profitable compared to non-vaccination; vaccinating cattle in a 1 year period provides substantial benefits to farmers (MRR = 9; BCR = 10.5) [67].

### Contagious bovine pleuropneumonia

Six studies assessed the profitability of interventions for controlling CBPP in cattle. The interventions include vaccination, antimicrobial treatment, surveillance, and a culling of infected animals at home. Except for culling, investments in vaccination, treatment or both treatment and vaccination, and surveillance, were all found to yield significant economic returns [68–71, 106, 117]. Average BCR for implementing a vaccination only strategy was 5.9 (range: 1.3 to 11.2). Average BCR for a jointly applied vaccination and antimicrobial treatment strategy was 2.2 (range: 2.0 to 2.4). Implementing vaccination, antimicrobial treatment and surveillance altogether provides a BCR of 1.3. However, a culling strategy is not profitable (BCR = 0.07).

### Contagious caprine pleuropneumonia

One study assessed the profitability of vaccination against CCPP infection in goats at different levels of vaccine efficacy [72]. BCR at 20% vaccine efficacy was 5.7, 50% was 27.2 and 95% was 61.9. Vaccination was thus profitable in all the scenarios assessed.

### Foot-and-mouth disease

Five studies evaluated the profitability of vaccination (*n* = 4) and antimicrobial treatment (*n* = 1) in controlling FMD in cattle. In all cases, the results showed that the investments in these interventions would yield high economic returns [46, 73, 74, 81, 107]. However, antimicrobial treatment of FMD lesions yielded higher economic returns (BCR = 33.6) compared to vaccination (BCR = 5.5 on average; range 1.9 to 11.5, IRR = 0.8).

### Lumpy skin disease

Two studies assessed the profitability of vaccination in controlling LSD in cattle [75, 76]. In both studies, vaccination was profitable; average MRR = 24.5 (range: 15.1 to 34), BCR = 25.6.

### Pasteurellosis

One study compared the profitability of implementing deworming or pasteurellosis vaccination in sheep. Only deworming was found to be profitable (BCR = 3.7) [49]. Another study compared the profitability of two vaccine formulations in controlling pasteurellosis; both vaccines were profitable (BCR = 4.7) [48].

### Peste des petits ruminants

Four studies [52, 68, 82, 83] evaluated the profitability of interventions aimed at controlling PPR in sheep and goats. All the interventions evaluated were cost-effective for controlling PPR, yielding significant economic returns on investment. Average BCR for controlling PPR by vaccination only was 14.0 (range: 12.4 to 15.6). Applying vaccination jointly with deworming provides a BCR of 3.1, while vaccination, surveillance and antimicrobial treatment applied jointly in PPR control yields a BCR of 2.1.

## Discussion

In this review, we aimed to summarize veterinary interventions implemented to control infectious diseases in ruminants in SSA, as well as their effectiveness in reducing the occurrence of diseases and deaths in livestock. Our review included both observational and experimental evaluations. Our results highlight vaccination as the main and currently dominant tool in the control of all the infectious diseases assessed. This could be due to the relative success of vaccination in the past as a control measure in eliminating several infectious diseases in livestock like foot-and-mouth disease and bluetongue in developed countries [119] as well as the infectious nature of the pathogens causing these diseases: viruses and bacteria. Particularly in the case of the global efforts against the highly virulent rinderpest disease which is the only animal disease to be eradicated globally after many years of devastative impact on animal health and productivity [120]. The other control measures including antimicrobial treatment, parasite control, test and slaughter, surveillance, and feed supplementation, were seldom used exclusively, but were usually combined with vaccination to achieve better results.

Our review showed that antimicrobials could limit disease progression in infected animals, thereby preventing further infection spread [56, 77, 98, 108, 115]. The test and slaughter strategy was also effective in the control of brucellosis and bovine TB [37, 43, 55, 88]. However, these control measures: antimicrobial treatment and, test and slaughter approaches, may not be feasible for effective disease control in the SSA region. They are either too expensive or impractical to implement, in the case of test and removal [121, 122], or lack effective regulation to achieve sustainable control, for antimicrobial treatment. Particularly in the case of antimicrobial treatment, a recent review of the PVS Pathway appraisals in African countries found that the veterinary services in 80% of countries in the region, had limited or in some cases no capability to administratively control the registration, import and production, distribution and usage of veterinary medicines and biologicals” [24]. Thus, the retail of antimicrobials are largely out of control, and antimicrobial treatment is widely practiced by farmers, without veterinary advice. Usage rates of antimicrobials range from 80 to 100% of farms in the region, with the main drugs in use being tetracyclines, aminoglycosides, and penicillin groups [123, 124]. Consequently, there is a significant concern about the safety of livestock products in the region, worsened by a lack of residue testing programmes in more than two-thirds of countries [24]. The high usage rates of antimicrobials coupled with a lack of testing could foster the development antimicrobial resistant pathogens.

Vaccination is currently without doubt, the main intervention tool for controlling infectious diseases in both humans and animals [125–127]. As noted in previous reviews [128–130], vaccination is highly effective in controlling most of the infectious diseases of interest in this review. However, given that a large proportion of the studies in our review (39%) were on-station clinical experiments, effectiveness under natural field conditions may be more limited due to extreme weather events, animal undernutrition and human error in vaccine administration among others. Under ideal conditions, different degrees of protection could be achieved by vaccination against specific pathogens, including protection against infection, disease progression and infection spread to other susceptible animals and humans [127]. The production of vaccines however is limited in SSA with only 17 countries producing vaccines in the region for livestock, mainly for local use in the countries [23]. About 20 different types of vaccines are produced in the region, a majority being vaccines for poultry especially against Newcastle disease. Vaccines produced for ruminant livestock are mainly against PPR, anthrax, and FMD. The production units are mostly small, with Ethiopia accounting for a large share of vaccines produced [23]. These vaccine production shortfalls coupled with huge challenges with distribution infrastructure in the region could affect farmers’ access to quality vaccines.

Good quality vaccines are key to any successful disease control strategy. Our review showed that some vaccines are less efficacious and in some cases, are even associated with increased risk of morbidity. While the negative effect of vaccination is difficult to explain, some bottlenecks have been identified to contribute to the reduction in effectiveness of vaccines under field conditions. For example, reasons for vaccination failures in this review were: potentially low vaccine efficacy due to over-attenuation [104] or pathogen resistance over time [47], loss of vaccine potency under unfavorable field conditions like adverse weather events [47, 50] and cold chain failure [58], and potential mismatch of circulating pathogen strain and the vaccines in use [60, 61, 65, 84, 86]. These setbacks are due mainly to poor handling of vaccines in the field [131], thus emphasizing the importance of the vehicle of vaccination delivery in the disease control strategy. More field evaluations of vaccine effectiveness in controlling livestock diseases under natural conditions are also needed. This will help to identify and address the challenges with deployment of vaccination in the field. There have been efforts to identify tools that minimize the field constraints associated with vaccination mobilization in SSA over the past decades. Some progress has been made in developing tools that address cold chain failures thus far. A good example is the recent development of an inexpensive locally produced passive cooling device that successfully maintained rabies vaccines under field conditions in rural Tanzania [132]. More tools such as this are needed to be scaled-up and deployed especially in rural settings in SSA, if the full dividends of vaccination are to be attained. Additionally, continued surveillance of the changes in the circulating pathogens through serotyping and subtyping as well as vaccine matching remains key to any successful control of infectious diseases [130].

Vaccination adoption and use by smallholder farmers and marginalized pastoral populations remain low in SSA. Factors accounting for this may be demand or supply driven. Significant weaknesses in the organizational structures of veterinary services particularly at the field level, is one of the major challenges identified by the review of PVS Pathway appraisals, as a supply side barrier in Africa. This is due mainly to human, financial and material resource constraints that hinder vaccine supplies and limits operational effectiveness [24]. The human resource capacity is estimated at an average of only seven animal health professionals (two veterinarians and five para-veterinarians) for every 100,000 inhabitants in SSA, compared to an average of 50, in countries like the United States and United Kingdom [23]. Thus, a stronger partnership with the private sector and donors would be required to address these supply side barriers in vaccination delivery [23, 24]. Demand side barriers are driven mainly by farmers’ loss of trust in the health services [133] or a lack of access to vaccination services due to the peculiar location of such communities [134]. Thus, strategies including awareness creation, improving vaccine supply, packaging and storage in the field have been proposed to increase vaccine adoption in developing countries [134]. Additionally, community engagement is also a valuable tool to addressing particularly demand side barriers linked to mistrust of health systems [133, 135]. Also, organizations including the Pan-African Veterinary Vaccine Center (PANVAC) remain crucial to the harmonization of disease control efforts in SSA through the setting of quality standards for animal vaccines [23].

Notwithstanding the benefits of vaccination, the question of its return on investment is particularly key for decision-making. Our review showed clearly that the application of vaccination as a disease control strategy is economically profitable regardless of whether it is implemented at the herd, community, or national levels. However, the profitability may depend on the pathogen, disease burden and quality of vaccines. For example, a test and slaughter strategy for controlling bovine TB in livestock would be more profitable [136], while vaccination of livestock is cost-effective in controlling PPR in livestock [68] and brucellosis in both livestock and humans [137]. Similar results of the cost-effectiveness of vaccination have been reported in other reviews in both human and animal studies [138, 139]. However, the approaches of the profitability analyses differ. The valuation of the cost-effectiveness of interventions in humans is based on non-monetary metrics, whereas in animals’ health, cost-effectiveness analysis is quantified in monetary metrics [140]. The profitability of vaccination as control strategy is understandable as vaccines generally decrease the incidence and severity of diseases thereby providing savings in the costs of measures previously used to deal with the disease, including costs of treatment or lost productivity and/or death of affected persons or animals. The sustainability of the funding mechanism for any disease control strategy is crucial, either with a free of cost or cost-recovery approach, to optimize the returns to investment. However, the choice of funding mechanisms should not be mutually exclusive; it should depend on the externalities involved for each peculiar disease (whether its control is for public or private good), and the capacity to pay [141]. The control of diseases that are transboundary in nature, including FMD, CBPP and PPR, must be treated as public good, with a greater share of the investment for their control financed from public sources. Thus, cost-effectiveness and willingness to pay studies on disease control strategies remain essential.

Vaccination could be even more effective and deliver high returns on investment if they could be combined with other strategies like surveillance and helminthic control, as our review revealed. Helminthic control have been shown to be largely effective in improving the productivity of livestock, and provides good returns on investment, particularly in small ruminants [142–144]. Uncontrolled helminthiases in livestock reduces appetite and antibody production, thereby negatively affecting their immune response to vaccination. Given that helminthiasis is a major problem affecting nutrition of livestock in SSA due to favorable environmental conditions, the inclusion of deworming as part of any disease control package would be both effective in improving animal health and provide good returns on investment, particularly in small ruminants as evident in this review [47, 49]. Other reviews have similarly highlighted the key role helminthic control plays in animal health and productivity, and proposed new tools to optimize the control efforts by addressing the problem of drug resistance [145, 146].

The anticipated improvement in livestock productivity with improved disease control in SSA may raise a sustainability concern with respect to the carbon footprint of livestock. Livestock-related contributions to methane emissions are relatively high; about 32% of all human activity related methane emissions [147]. Thus, the livestock sector must also reduce its emissions as part of global efforts to mitigate climate change. But having highly productive livestock, would effectively result in producing the required nutritional requirements of the population with fewer animals [148]. We argue that to achieve sustainability and enhance the reduction of greenhouse emissions in livestock, infectious diseases must be controlled effectively. If livestock are largely healthy, fewer animals would be required for food-producing purposes [149]. This phenomenon could be likened to the population dynamics during the demographic transition, where a sustained decline in mortality was the precondition for families to reduce their fertility, no longer needing to have more children than needed in anticipation of losing some children to diseases [150]. Moreover, the largely extensive nature of the livestock production system in SSA makes it less dependent on feeding animals with human-edible crops with its attendant loss of biodiversity. Nevertheless, to achieve sustainability in livestock production, in an effective disease control regime, there would be a need for strict land and grassland use controls that would optimize the inputs and outputs in the production of livestock.

Our review had some challenges; the differences in the outcomes of interest or the measure of intervention effectiveness and/or profitability in some of the studies did not allow us to derive a pooled estimate of effectiveness and/ or profitability in all cases. In addition, as the focus of the review was to map the scope of evidence in the literature on what preventive interventions are applied, their effectiveness and/or profitability, an assessment of methodological limitations in the included studies was not done [29]. It would be interesting to stratify the interventions’ effectiveness and profitability by farming system. However, the unavailability of this information in included studies did not permit such analysis. Our review focused on interventions for which reduction in infectious livestock disease occurrence or deaths was a directly measurable outcome or could be inferred indirectly from another reported outcome. Thus, for studies that did not report protective rates or BCRs of the interventions, but had data on morbidity and/or mortality in intervention and control groups, or intervention and disease costs, we were able to compute protection rates and BCRs based on the data published to allow for a comparison of intervention effectiveness and profitability across studies. This review thus, has provided good evidence of the value of veterinary interventions applied in controlling infectious diseases in SSA, in spite of these limitations. Future reviews would benefit from having standardized measures of assessing effectiveness and profitability of interventions in original research articles. It is clear however, that profitability analyses of controlling some of the infectious diseases are lacking. More studies on profitability of control strategies therefore are needed.

## Conclusion

This review shows that vaccination is currently the main strategy for controlling infectious diseases in livestock in SSA. Other strategies such as test and removal or antimicrobial treatment appear more challenging in the resource constrained and less regulated settings of SSA. Helminthic control, particularly in small ruminants, also appears to be effective in improving productivity and profitability of livestock when combined with vaccination. Despite their potential effectiveness and high returns on investment of vaccination as a control measure, factors such as adverse weather events, cold chain failure, and poor surveillance of circulating pathogen strains, could cause vaccines to be ineffective in practice. To achieve effective control of infectious livestock diseases in SSA, vaccination strategies should ideally integrate deworming and continuous surveillance capable of identifying new pathogens of interest. Optimal vaccine delivery tools may also help to minimize the impact of unfavorable field conditions, while maximizing the impact of the control strategy.

## Supplementary Information

**Additional file 1.** Search terms used on PUBMED, SCOPUS and African Journals Online.

**Additional file 2: Table S1.** Overview of the studies reviewed.

## Data Availability

All data generated or analyzed during this study are included in this published article [and its supplementary information files].
