# Supplementary material for: Effectiveness and profitability of preventive veterinary interventions in controlling infectious diseases of ruminant livestock in sub-Saharan Africa: a scoping review
Source: BMC Vet Res. 2022 Sep 2;18:332. doi: 10.1186/s12917-022-03428-9 (PMC9438146; doi:10.1186/s12917-022-03428-9)
Supplement: Supplementary file 1 — Additional file 1. Search terms used on PUBMED, SCOPUS and African Journals Online. [file 12917_2022_3428_MOESM1_ESM.docx]

**Additional file 1: Search terms used on PUBMED, SCOPUS and African Journals Online**

Additional file 1 provides the search terms used for each of the database searches conducted.

**PUBMED (1842 hits)**

("Africa South of the Sahara"[Mesh] OR Angola[Mesh:NoExp] OR Benin[Mesh:NoExp] OR Botswana[Mesh:NoExp] OR "Burkina Faso"[Mesh:NoExp] OR Burundi[Mesh:NoExp] OR Cameroon[Mesh:NoExp] OR "Cabo Verde"[Mesh:NoExp] OR "Central African Republic"[Mesh:NoExp] OR Chad[Mesh:NoExp] OR Comoros[Mesh:NoExp] OR Congo[Mesh:NoExp] OR "Cote d'Ivoire"[Mesh:NoExp] OR Djibouti[Mesh:NoExp] OR "Equatorial Guinea"[Mesh:NoExp] OR Eritrea[Mesh:NoExp] OR Ethiopia[Mesh:NoExp] OR Gabon[Mesh:NoExp] OR Gambia[Mesh:NoExp] OR Ghana[Mesh:NoExp] OR Guinea[Mesh:NoExp] OR Guinea-Bissau[Mesh:NoExp] OR Kenya[Mesh:NoExp] OR Lesotho[Mesh:NoExp] OR Liberia[Mesh:NoExp] OR Madagascar[Mesh:NoExp] OR Malawi[Mesh:NoExp] OR Mali[Mesh:NoExp] OR Mauritania[Mesh:NoExp] OR Mauritius[Mesh:NoExp] OR Mozambique[Mesh:NoExp] OR Namibia[Mesh:NoExp] OR Niger[Mesh:NoExp] OR Nigeria[Mesh:NoExp] OR Rwanda[Mesh:NoExp] OR "Sao Tome and Principe"[Mesh:NoExp] OR Senegal[Mesh:NoExp] OR Seychelles[Mesh:NoExp] OR "Sierra Leone"[Mesh:NoExp] OR Somalia[Mesh:NoExp] OR "South Africa"[Mesh:NoExp] OR "South Sudan"[Mesh:NoExp] OR Sudan[Mesh:NoExp] OR Swaziland[Mesh:NoExp] OR Tanzania[Mesh:NoExp] OR Togo[Mesh:NoExp] OR Uganda[Mesh:NoExp] OR Congo[Mesh:NoExp] OR Zaire[Mesh:NoExp] OR Zambia[Mesh:NoExp] OR Zimbabwe[Mesh:NoExp] OR ((Angola[tiab] OR Benin[tiab] OR Botswana[tiab] OR Bobo-Dioulasso[tiab] OR "Burkina Faso"[tiab] OR Burundi[tiab] OR Cameroon[tiab] OR "Cabo Verde"[tiab] OR "Central African Republic"[tiab] OR Chad[tiab] OR Comoros[tiab] OR Congo[tiab] OR "Cote d'Ivoire"[tiab] OR Djibouti[tiab] OR "Equatorial Guinea"[tiab] OR Eritrea[tiab] OR Ethiopia[tiab] OR Gabon[tiab] OR Gambia[tiab] OR Ghana[tiab] OR Guinea[tiab] OR Guinea-Bissau[tiab] OR Kenya[tiab] OR Lesotho[tiab] OR Liberia[tiab] OR Madagascar[tiab] OR Malawi[tiab] OR Mali[tiab] OR Mauritania[tiab] OR Mauritius[tiab] OR Mozambique[tiab] OR Namibia[tiab] OR Niger[tiab] OR Nigeria[tiab] OR Rwanda[tiab] OR "Sao Tome and Principe"[tiab] OR Senegal[tiab] OR Seychelles[tiab] OR "Sierra Leone"[tiab] OR Somalia[tiab] OR "South Africa"[tiab] OR "South Sudan"[tiab] OR Sudan[tiab] OR Swaziland[tiab] OR Tanzania[tiab] OR Togo[tiab] OR Uganda[tiab] OR Zaire[tiab] OR Zambia[tiab] OR Zimbabwe[tiab] OR "Africa South of the Sahara"[tiab] OR "Sub-Saharan Africa"[tiab]))) AND (ruminant[Mesh] OR livestock[Mesh] OR ruminant*[tiab] OR Antelope*[tiab] OR Hippotragine[tiab] OR Buffalo*[tiab] OR Bubalus[tiab] OR Syncerus[tiab] OR Bison[tiab] OR Cattle[tiab] OR Cow[tiab] OR Zebu[tiab] OR Yak[tiab] OR Bos[tiab] OR Deer[tiab] OR Giraffe*[tiab] OR Okapi[tiab] OR Goat[tiab] OR Capra[tiab] OR Sheep[tiab] OR Ovis[tiab] OR Mouflon[tiab]) AND ("Animal Disease*"[Mesh] OR "Abortion, Veterinary"[tiab] OR Brucellosis[tiab] OR "Bang Disease"[tiab] OR Tuberculos*[tiab] OR Mycobacteri*[tiab] OR "Foot-and-Mouth Disease"[tiab] OR Blackleg[tiab] OR "Clostridium chauvoei"[tiab] OR Anthrax[tiab] OR "Bacillus anthracis Infection*"[tiab] OR "Sheep Pox"[tiab] OR "Goat Pox"[tiab] OR Capripoxvirus[tiab] OR "Pox Virus"[tiab] OR Peste-des-Petits-Ruminants[tiab] OR Pseudorinderpest[tiab] OR Pleuropneumonia*[tiab] OR "Lumpy Skin Disease"[tiab] OR Pasteurellosis[tiab] OR "Shipping Fever"[tiab]) AND ("Public health practice"[Mesh] OR "Communicable Disease Control"[tiab] OR "Animal Culling"[tiab] OR "Disease Notification"[tiab] OR Fumigation[tiab] OR Immunization[tiab] OR Vaccination[tiab] OR "Infection Control"[tiab] OR "Mandatory Testing"[tiab] OR "Mass Drug Administration"[tiab] OR "Contact Tracing"[tiab] OR "Physical Distancing"[tiab] OR Quarantine[tiab] OR "Universal Precautions"[tiab] OR Disinfection[tiab] OR Decontamination[tiab] OR "Mass Screening"[tiab] OR "Primary Prevention"[tiab] OR "Quaternary Prevention"[tiab] OR "Secondary Prevention"[tiab] OR "Tertiary Prevention"[tiab] OR prevent*[tiab] OR reduc*[tiab])

**SCOPUS (906 hits)**

(INDEXTERMS("Africa South of the Sahara") OR INDEXTERMS("Angola") OR INDEXTERMS("Benin") OR INDEXTERMS("Botswana") OR INDEXTERMS("Burkina Faso") OR INDEXTERMS("Burundi") OR INDEXTERMS("Cameroon") OR INDEXTERMS("Cabo Verde") OR INDEXTERMS("Central African Republic") OR INDEXTERMS("Chad") OR INDEXTERMS("Comoros") OR INDEXTERMS("Congo") OR INDEXTERMS("Cote d'Ivoire") OR INDEXTERMS("Djibouti") OR INDEXTERMS("Equatorial Guinea") OR INDEXTERMS("Eritrea") OR INDEXTERMS("Ethiopia") OR INDEXTERMS("Gabon") OR INDEXTERMS("Gambia") OR INDEXTERMS("Ghana") OR INDEXTERMS("Guinea") OR INDEXTERMS("Guinea-Bissau") OR INDEXTERMS("Kenya") OR INDEXTERMS("Lesotho") OR INDEXTERMS("Liberia") OR INDEXTERMS("Madagascar") OR INDEXTERMS("Malawi") OR INDEXTERMS("Mali") OR INDEXTERMS("Mauritania") OR INDEXTERMS("Mauritius") OR INDEXTERMS("Mozambique") OR INDEXTERMS("Namibia") OR INDEXTERMS("Niger") OR INDEXTERMS("Nigeria") OR INDEXTERMS("Rwanda") OR INDEXTERMS("Sao Tome and Principe") OR INDEXTERMS("Senegal") OR INDEXTERMS("Seychelles") OR INDEXTERMS("Sierra Leone") OR INDEXTERMS("Somalia") OR INDEXTERMS("South Africa") OR INDEXTERMS("South Sudan") OR INDEXTERMS("Sudan") OR INDEXTERMS("Swaziland") OR INDEXTERMS("Tanzania") OR INDEXTERMS("Togo") OR INDEXTERMS("Uganda") OR INDEXTERMS("Congo") OR INDEXTERMS("Zaire") OR INDEXTERMS("Zambia") OR INDEXTERMS("Zimbabwe") OR ((TITLE-ABS("Angola") OR TITLE-ABS("Benin") OR TITLE-ABS("Botswana") OR TITLE-ABS("Bobo-Dioulasso") OR TITLE-ABS("Burkina Faso") OR TITLE-ABS("Burundi") OR TITLE-ABS("Cameroon") OR TITLE-ABS("Cabo Verde") OR TITLE-ABS("Central African Republic") OR TITLE-ABS("Chad") OR TITLE-ABS("Comoros") OR TITLE-ABS("Congo") OR TITLE-ABS("Cote d'Ivoire") OR TITLE-ABS("Djibouti") OR TITLE-ABS("Equatorial Guinea") OR TITLE-ABS("Eritrea") OR TITLE-ABS("Ethiopia") OR TITLE-ABS("Gabon") OR TITLE-ABS("Gambia") OR TITLE-ABS("Ghana") OR TITLE-ABS("Guinea") OR TITLE-ABS("Guinea-Bissau") OR TITLE-ABS("Kenya") OR TITLE-ABS("Lesotho") OR TITLE-ABS("Liberia") OR TITLE-ABS("Madagascar") OR TITLE-ABS("Malawi") OR TITLE-ABS("Mali") OR TITLE-ABS("Mauritania") OR TITLE-ABS("Mauritius") OR TITLE-ABS("Mozambique") OR TITLE-ABS("Namibia") OR TITLE-ABS("Niger") OR TITLE-ABS("Nigeria") OR TITLE-ABS("Rwanda") OR TITLE-ABS("Sao Tome and Principe") OR TITLE-ABS("Senegal") OR TITLE-ABS("Seychelles") OR TITLE-ABS("Sierra Leone") OR TITLE-ABS("Somalia") OR TITLE-ABS("South Africa") OR TITLE-ABS("South Sudan") OR TITLE-ABS("Sudan") OR TITLE-ABS("Swaziland") OR TITLE-ABS("Tanzania") OR TITLE-ABS("Togo") OR TITLE-ABS("Uganda") OR TITLE-ABS("Zaire") OR TITLE-ABS("Zambia") OR TITLE-ABS("Zimbabwe") OR TITLE-ABS("Africa South of the Sahara") OR TITLE-ABS("Sub-Saharan Africa")))) AND (INDEXTERMS("ruminant") OR INDEXTERMS("livestock") OR TITLE-ABS("ruminant*") OR TITLE-ABS("Antelope*") OR TITLE-ABS("Hippotragine") OR TITLE-ABS("Buffalo*") OR TITLE-ABS("Bubalus") OR TITLE-ABS("Syncerus") OR TITLE-ABS("Bison") OR TITLE-ABS("Cattle") OR TITLE-ABS("Cow") OR TITLE-ABS("Zebu") OR TITLE-ABS("Yak") OR TITLE-ABS("Bos") OR TITLE-ABS("Deer") OR TITLE-ABS("Giraffe*") OR TITLE-ABS("Okapi") OR TITLE-ABS("Goat") OR TITLE-ABS("Capra") OR TITLE-ABS("Sheep") OR TITLE-ABS("Ovis") OR TITLE-ABS("Mouflon")) AND (INDEXTERMS("Animal Disease*") OR TITLE-ABS("Abortion, Veterinary") OR TITLE-ABS("Brucellosis") OR TITLE-ABS("Bang Disease") OR TITLE-ABS("Tuberculos*") OR TITLE-ABS("Mycobacteri*") OR TITLE-ABS("Foot-and-Mouth Disease") OR TITLE-ABS("Blackleg") OR TITLE-ABS("Clostridium chauvoei") OR TITLE-ABS("Anthrax") OR TITLE-ABS("Bacillus anthracis Infection*") OR TITLE-ABS("Sheep Pox") OR TITLE-ABS("Goat Pox") OR TITLE-ABS("Capripoxvirus") OR TITLE-ABS("Pox Virus") OR TITLE-ABS("Peste-des-Petits-Ruminants") OR TITLE-ABS("Pseudorinderpest") OR TITLE-ABS("Pleuropneumonia*") OR TITLE-ABS("Lumpy Skin Disease") OR TITLE-ABS("Pasteurellosis") OR TITLE-ABS("Shipping Fever")) AND (INDEXTERMS("Public health practice") OR TITLE-ABS("Communicable Disease Control") OR TITLE-ABS("Animal Culling") OR TITLE-ABS("Disease Notification") OR TITLE-ABS("Fumigation") OR TITLE-ABS("Immunization") OR TITLE-ABS("Vaccination") OR TITLE-ABS("Infection Control") OR TITLE-ABS("Mandatory Testing") OR TITLE-ABS("Mass Drug Administration") OR TITLE-ABS("Contact Tracing") OR TITLE-ABS("Physical Distancing") OR TITLE-ABS("Quarantine") OR TITLE-ABS("Universal Precautions") OR TITLE-ABS("Disinfection") OR TITLE-ABS("Decontamination") OR TITLE-ABS("Mass Screening") OR TITLE-ABS("Primary Prevention") OR TITLE-ABS("Quaternary Prevention") OR TITLE-ABS("Secondary Prevention") OR TITLE-ABS("Tertiary Prevention") OR TITLE-ABS("prevent*") OR TITLE-ABS("reduc*"))

**African Journals Online (179 hits)**

[(ruminant OR livestock OR Antelope OR Hippotragus OR Buffalo OR Bubalus OR Bison OR Cattle OR Cow OR Zebu OR Yak OR Bos OR Deer OR Giraffe OR Okapi OR Goat OR Capra OR Sheep OR Ovis OR Mouflon) AND (Animal Disease* OR Abortion OR Brucellosis OR Bang Disease OR Tuberculos* OR Mycobacter* OR Foot-and-Mouth Disease OR Black Leg OR Clostridium Chauvoei OR Anthrax OR Bacillus anthracis infection OR Sheep Pox OR Goat Pox OR Capripoxvirus OR Pox Virus OR Peste-des-Petits-Ruminants OR Pseudo Rinderpest OR Pleuropneumonia OR Lumpy Skin Disease OR Pasteurellosis OR Shipping Fever) AND (Public health practice OR Communicable Disease Control OR Animal Culling OR Disease Notification OR Fumigation OR Immunization OR Vaccination OR Infection Control OR Mandatory Testing OR Mass Drug Administration OR Contact Tracing OR Physical Distancing OR Quarantine OR Universal Precautions OR Disinfection OR Decontamination OR Mass Screening OR Primary Prevention OR Quaternary Prevention OR Secondary Prevention OR Tertiary Prevention OR prevent* OR reduce*)]
