# Supplementary material for: Effectiveness and profitability of preventive veterinary interventions in controlling infectious diseases of ruminant livestock in sub-Saharan Africa: a scoping review
Source: BMC Vet Res. 2022 Sep 2;18:332. doi: 10.1186/s12917-022-03428-9 (PMC9438146; doi:10.1186/s12917-022-03428-9)
Supplement: Supplementary file 2 — Additional file 2: Table S1. Overview of the studies reviewed. [file 12917_2022_3428_MOESM2_ESM.docx]

**Table S1: Overview of the studies reviewed**

The table details the characteristics of the included studies in addition to the references in parenthesis and reference list at the end, as captured in the manuscript

| **Author (references)** | **Year** | **Country** | **Objective of study** | **Study design** | **Data sources** |
| --- | --- | --- | --- | --- | --- |
| Admassu B. et al. (36) | 2005 | Ethiopia | Impact assessment of CAHWs work on disease prevalence in cattle, sheep and goats | Cross-sectional study (participatory) | FGDs & interviews |
| Alhaji NB. et al. (111) | 2020 | Nigeria | Cost-benefits of FMD control in pastoral local dairy cattle production systems | Cross-sectional study (survey) | Questionnaire |
| Alhaji NB. Et al. (107) | 2017 | Nigeria | Cost-effectiveness of vaccination against CBPP infection in cattle | Cross-sectional study (survey) | Questionnaire & interviews |
| Ameni G. et al. (46) | 2007 | Ethiopia | Effectiveness of test and segregation on Bovine TB incidence in cattle | Experimental study (field trial) | Physical & laboratory examination |
| Ameni G. et al. (42) | 2018 | Ethiopia | Efficacy of vaccination against Bovine TB infection in calves | Experimental study (clinical trial) | Physical & laboratory examination |
| Ameni G. et al. (41) | 2010 | Ethiopia | Effectiveness of vaccination against Bovine TB infection in neonatal calves | Experimental study (clinical trial) | Physical & laboratory examination |
| Anderson EC. et al. (82) | 1974 | Kenya | Compare prevalence of FMD infection in cattle from vaccinated and unvaccinated areas | Cross-sectional study (survey) | Laboratory examination |
| Awa DN. et al. (99) | 2000 | Cameroon | Effectiveness and cost-benefits of deworming and PPR vaccination in goats and sheep | Experimental study (field trial) | Questionnaire |
| Ayele B. et al. (103) | 2016 | Ethiopia | Cost-benefits of vaccination against blackleg infection in cattle | Cross-sectional (survey, participatory) | Questionnaire & FGDs |
| Ayelet G. et al. (90) | 2013 | Ethiopia | Compare prevalence of LSD infection and deaths in vaccinated and unvaccinated cattle | Cross-sectional study (survey) | Questionnaire, lab & physical examination |
| Ayelet G. et al. (91) | 2014 | Ethiopia | Compare prevalence of LSD infection and deaths in vaccinated and unvaccinated cattle | Cross-sectional study Retrospective study | Questionnaire, data review (secondary) |
| Ba SB. et al. (34) | 1996 | Mali | Effectiveness of vaccination and deworming on mortality in goats | Experimental study (field trial) | Questionnaire, lab examination |
| Barasa M. et al. (112) | 2008 | South Sudan | Cost-benefits of vaccination against FMD in cattle | Cross-sectional (participatory) | Interviews, literature review, observation |
| Berggren SA. (44) | 1981 | Malawi | Effectiveness of vaccination against Bovine TB incidence and spread in cattle | Experimental study (field trial) | Questionnaire, lab & physical examination |
| Berggren SA. (45) | 1977 | Malawi | Compare incidence of Bovine TB infection in vaccinated and unvaccinated cattle | Cross-sectional study (survey) | Questionnaire & physical examination |
| Bertram MR. et al. (83) | 2018 | Cameroon | Effectiveness of vaccination against FMD infection in cattle over 1 year | Experimental study (field trial) | Observation & lab examination |
| Camus E. (37) | 1980 | Côte d'Ivoire | Effectiveness of vaccination against abortions and still births in cattle due to Brucellosis | Experimental study (field trial) | Questionnaire, data review & observation |
| Catley A. et al. (35) | 2009 | Ethiopia | Effectiveness of vaccination against cattle, goat and sheep mortality in normal and drought years | Cross-sectional study (participatory), Retrospective study | Questionnaire, Interviews, data review, observation |
| Caufour P. et al. (85) | 2014 | Ethiopia | Efficacy of vaccination against Capripox and PPR in goats with pre-existing immunity | Experimental study (clinical trial) | Observation, lab & physical examination |
| Chema S. (80) | 1975 | Kenya | Effectiveness and cost-benefits of vaccination against occurrence of FMD outbreaks in cattle | Experimental study (before and after) | Observation, data review & lab tests |
| Cloete M. et al. (75) | 2008 | South Africa | Efficacy of different vaccine formulations against FMD infection in cattle | Experimental study (clinical trial) | Physical & laboratory examination |
| Coackley W. & Weston SJ. (39) | 1957 | Kenya | Efficacy of vaccination against blackleg deaths in cattle and sheep | Experimental study (clinical & field trial) | Data review, physical & lab examination |
| Domenech J. (55) | 1979 | Ethiopia | Compare the prevalence of CBPP infection in cattle at different vaccination coverage levels | Cross-sectional study (survey) | Data review & lab examination |
| Doutre MP. & Chambron J. (68) | 1970 | Senegal | Efficacy of vaccination against CBPP deaths in cattle at 3, 7 and 14 months post-exposure | Experimental study (clinical trial) | Questionnaire, lab tests & observation |
| Doutre MP. et al. (69) | 1972 | Senegal | Efficacy of vaccination against CBPP infection in cattle | Experimental study (clinical trial) | Questionnaire, lab tests & observation |
| ElArbi AS. et al. (118) | 2019 | Mauritania | Cost-benefits of different vaccination strategies in controlling PPR in sheep and goats | Cross-sectional study (surveys) | Questionnaire, data review |
| Ellwood DE. & Waddington FG. (43) | 1972 | Malawi | Efficacy of vaccination against Bovine TB infection and spread of lung lesions in cattle | Experimental study (clinical trial) | Observation, lab & physical examination |
| Emslie FR. & Nel JR. (38) | 2002 | South Africa | Effectiveness of a test and slaughter strategy on Brucellosis infection in goat and sheep | Experimental study (field trial) | Data review & lab examination |
| Fadiga M. et al. (105) | 2013 | Nigeria | Cost-benefits of targeted interventions in the control of CBPP & PPR in cattle, sheep & goats | Cross-sectional study Retrospective study | Interviews, FGDs, data review |
| Garba SA. et al. (67) | 1989 | Nigeria | Efficacy of different vaccine formulations against CBPP infection and deaths in cattle | Experimental study (clinical trial) | Observation, lab & physical examination |
| Gari G. et al. (88) | 2015 | Ethiopia | Efficacy of different vaccine formulations against LSD infection in cattle | Experimental study (clinical trial) | Observation, lab & physical examination |
| Gari G. et al. (115) | 2011 | Ethiopia | Cost-benefits of vaccination in controlling LSD in cattle | Cross-sectional (survey) | Questionnaire, data review & interviews |
| Gilbert FR. & Windsor RS. (63) | 1971 | Kenya | Efficacy of different vaccine formulations against CBPP infection in cattle | Experimental study (clinical trial) | Observation, lab & physical examination |
| Gilbert FR. et al. (62) | 1970 | Kenya | Efficacy of vaccination against CBPP infection in cattle | Experimental study (clinical trial) | Observation, lab & physical examination |
| Gray MA. et al. (64) | 1986 | Kenya | Efficacy of different vaccine formulations against CBPP infection and deaths in cattle | Experimental study (clinical trial) | Observation, lab & physical examination |
| Gummow B. & Mapham PH. (93) | 2000 | South Africa | Effectiveness and cost-benefits of different vaccination formulations against Pasteurellosis infection and deaths in cattle | Experimental study (field trial) | Observation, data review & physical examination |
| Holzer B. et al. (95) | 2016 | Kenya | Efficacy of different vaccine formulations against PPR infection in goats | Experimental study (clinical trial) | Observation & lab examination |
| Hübschle OJ. et al. (54) | 2003 | Namibia | Efficacy of vaccination against CBPP deaths in cattle | Experimental study (clinical trial) | Observation, lab & physical examination |
| Huebschle OJB. et al. (50) | 2006 | Namibia | Efficacy of antimicrobial treatment against CBPP infection and spread in cattle | Experimental study (clinical trial) | Observation, lab & physical examination |
| Jarikre TA. et al. (96) | 2019 | Nigeria | Efficacy of different vaccine formulations against PPR infection in goats | Experimental study (clinical trial) | Observation, lab & physical examination |
| Jemberu WT. et al. (79) | 2020 | Ethiopia | Effectiveness of vaccination against FMD infection in cattle | Experimental study (field trial) | Observation, interview & questionnaire |
| Jemberu WT. et al. (113) | 2016 | Ethiopia | Cost-benefits of different vaccination strategies in controlling FMD in cattle | Retrospective study (secondary data) | Literature and data review |
| Kairu-Wanyoike SW. et al. (108) | 2014 | Kenya | Cost-benefits of vaccination in controlling CBPP in cattle | Cross-sectional study (survey) | Questionnaire |
| Kairu-Wanyoike SW. et al. (106) | 2017 | Kenya | Cost-benefits of vaccination in controlling CBPP in cattle at household and community levels | Cross-sectional study Retrospective study Longitudinal study | Questionnaire, data review & interviews |
| Lancelot R. et al. (92) | 2002 | Senegal | Effectiveness of deworming and vaccination on mean duration of survival of goats within 1 year | Experimental study (field trial) | Questionnaire & physical examination |
| Lazarus DD. et al. (78) | 2020 | South Africa | Efficacy of different vaccination doses against FMD infection in goats | Experimental study (clinical trial) | Observation, lab & physical examination |
| le Roex N. et al. (48) | 2016 | South Africa | Effectiveness of a test and cull program against Bovine TB infection in Buffalos | Experimental study (clinical trial) | Questionnaire, lab & physical examination |
| Lendzele SS. et al. (74) | 2020 | Cameroon | Efficacy of Tri-Solfen therapy vs antimicrobial treatment on FMD wound healing in cattle | Experimental study (clinical trial) | Observation, physical exam, questionnaire |
| Lesnoff M. et al. (94) | 2000 | Senegal | Effectiveness and cost-benefits of deworming and vaccination against Pasteurellosis infection in sheep | Experimental study (field trial) | Questionnaire, observation & physical examination |
| Lindley EP. (72) | 1967 | Sudan | Efficacy of vaccination against development of CBPP lesions in cattle | Experimental study (clinical trial) | Observation, lab & physical examination |
| Lyons NA. et al. (81) | 2015 | Kenya | Effectiveness of vaccination against incidence of FMD infection in cattle | Longitudinal study (cohort) | Observation, physical exam, questionnaire |
| Maree FF. et al. (76) | 2015 | South Africa | Efficacy of two different vaccine formulations against FMD infection in cattle | Experimental study (clinical trial) | Observation, lab & physical examination |
| Mariner JC. et al. (71) | 2006 | Sudan, Tanzania | Effectiveness of different vaccination strategies against CBPP infection and deaths in cattle | Cross-sectional study Retrospective study | FGDs, interviews & literature review |
| Mariner JC. et al. (70) | 2006 | Kenya, Sudan, Tanzania | Effectiveness of vaccination against CBPP infection and deaths in cattle | Retrospective study (secondary data) | Literature and data review |
| Martrenchar A. et al. (86) | 1997 | Cameroon | Efficacy of vaccination against PPR and Goat pox infection and deaths in goats | Experimental study (clinical trial) | Observation, lab & physical examination |
| Martrenchar A. et al. (98) | 1999 | Cameroon | Efficacy of vaccination against PPR deaths in goats and sheep | Experimental study (field trial) | Observation, physical exam, questionnaire |
| Masiga WN. (65) | 1972 | Kenya | Efficacy of vaccination against CBPP infection and deaths in different cattle breeds | Experimental study (clinical trial) | Observation, lab & physical examination |
| Molla W. et al. (116) | 2017 | Ethiopia | Cost-benefits of vaccination in controlling LSD infection in cattle | Cross-sectional study (survey) | Questionnaire |
| Molla W. et al. (89) | 2017 | Ethiopia | Effectiveness of vaccination against LSD infection in cattle | Experimental study Cross-sectional study | Questionnaire, lab & physical examination |
| Muuka G. et al. (52) | 2019 | Kenya, Zambia | Efficacy of different antimicrobial treatments against CBPP lung lesion resolution in cattle | Experimental study (clinical trial) | Observation, lab & physical examination |
| Mwacalimba KK. et al. (102) | 2013 | Zambia | Cost-benefits of a test and slaughter strategy in controlling Bovine TB in cattle | Retrospective study (secondary data) | Literature and data review |
| Ngichabe CK. et al. (87) | 2002 | Kenya | Efficacy of vaccination against LSD infection in cattle | Experimental study (clinical trial) | Observation, lab & physical examination |
| Niang M. et al. (51) | 2010 | Mali | Efficacy of antimicrobial treatment against CBPP infection and deaths in cattle | Experimental study (clinical trial) | Observation, lab & physical examination |
| Njoya A. et al. (100) | 2005 | Cameroon | Effectiveness of food supplementation and prophylaxis against PPR on deaths in sheep | Experimental study (field trial) | Observation, physical exam, questionnaire |
| Nkando I. et al. (59) | 2012 | Kenya | Efficacy of different vaccine formulations against CBPP lung pathology in cattle | Experimental study (clinical trial) | Observation, lab & physical examination |
| Nkando I. et al. (60) | 2016 | Kenya | Efficacy of different vaccine formulations against CBPP infection in cattle | Experimental study (clinical trial) | Observation, lab & physical examination |
| Nyaguthii DM. et al. (84) | 2019 | Kenya | Compare prevalence of FMD infection in vaccinated and unvaccinated cattle | Cross-sectional study (survey) | Questionnaire |
| Onono JO. et al. (109) | 2014 | Kenya | Cost-benefits of different intervention packages in controlling CBPP in cattle | Cross-sectional study Retrospective study | Interviews & literature review |
| Renault V. et al. (110) | 2019 | Kenya | Cost-benefits of vaccination in controlling CCPP in goats | Longitudinal study (cohort) | Questionnaire, data review & observation |
| Reynolds L. & Francis PA. (101) | 1988 | Nigeria | Effectiveness of dipping and vaccination against PPR on kidding and deaths in goats | Experimental study (field trial) | Observation, data review, questionnaire |
| Roug A. et al. (47) | 2014 | Tanzania | Effectiveness of a test and removal strategy on Bovine TB prevalence in cattle | Retrospective study (secondary data) | Observations, data & literature review |
| Rurangirwa FR. et al. (73) | 1991 | Kenya | Efficacy of vaccination against CCPP infection and deaths in goats | Experimental study (field trial) | Observation, lab & physical examination |
| Scott KA. et al. (77) | 2017 | South Africa | Efficacy of different vaccine formulations against FMD infection in cattle | Experimental study (clinical trial) | Observation, lab & physical examination |
| Souley Kouato B. et al. (114) | 2018 | Niger | Cost-benefits of vaccination in controlling FMD infection in cattle | Retrospective study (secondary data) | Data review |
| Stem C. (117) | 1993 | Niger | Cost-benefits of vaccination in controlling PPR infection in goats | Retrospective study (secondary data) | Observations, data & literature review |
| Suleiman A. et al. (56) | 2015 | Nigeria | Compare the prevalence of CBPP infection in vaccinated and unvaccinated cattle | Cross-sectional study (survey) | Questionnaire & lab examination |
| Tambi NE. et al. (104) | 2006 | Multiple countries (12)* | Cost-benefits of vaccination and treatment in controlling CBPP infection in cattle | Retrospective study (secondary data) | Data & literature review |
| Tambuwal F. & Egwu G. (58) | 2011 | Nigeria | Compare the prevalence of CBPP infection in cattle at different vaccination coverage levels | Retrospective study (secondary data) | Data review |
| Thiaucourt F. et al. (53) | 2004 | Cameroon, Kenya | Efficacy of different vaccine formulations and antimicrobial treatment against CBPP infection | Experimental study (clinical trial) | Observation, lab & physical examination |
| Thiaucourt F. et al. (61) | 2000 | Kenya,Namibia Cameroon | Efficacy of vaccination against CBPP infection and deaths in cattle | Experimental study (clinical trial) | Observation, lab & physical examination |
| Waddington FG. & Ellwood DC. (40) | 1972 | Malawi | Efficacy of vaccination against Bovine TB infection in cattle | Experimental study (clinical trial) | Observation, lab & physical examination |
| Wesonga H. & Thiaucourt F. (66) | 2000 | Kenya | Efficacy of different vaccine formulations against CBPP infection in cattle | Experimental study (clinical trial) | Observation, lab & physical examination |
| Wosu LO. et al. (97) | 1990 | Nigeria | Effectiveness of vaccination across different seasons against PPR infection in goats | Experimental study (field trial) | Observation, physical examination |
| Zerbo LH. et al. (57) | 2021 | Burkina Faso | Compare the prevalence of CBPP infection in cattle at different vaccination coverage levels | Cross-sectional study (survey) | Questionnaire & lab examination |

***Countries**: Burkina Faso, Chad, Côte d'Ivoire, Ethiopia, Ghana, Guinea, Kenya, Mali, Mauritania, Niger, Tanzania, Uganda

**Reference list**

The reference list is presented in alphabetical order with numbered references as in manuscript text

36. Admassu B, Nega S, Haile T, Abera B, Hussein A, Catley A. Impact assessment of a community-based animal health project in Dollo Ado and Dollo Bay districts, southern Ethiopia. Trop Anim Health Prod. 2005;37(1):33-48.

73. Alhaji NB, Amin J, Aliyu MB, Mohammad B, Babalobi OO, Wungak Y, et al. Economic impact assessment of foot-and-mouth disease burden and control in pastoral local dairy cattle production systems in Northern Nigeria: A cross-sectional survey. Prev Vet Med. 2020;177:104974.

70. Alhaji NB, Babalobi OO. Economic impacts assessment of pleuropneumonia burden and control in pastoral cattle herds of north-central Nigeria. Bulletin of Animal Health and Production in Africa. 2017;65(2):235-48.

55. Ameni G, Aseffa A, Sirak A, Engers H, Young DB, Hewinson RG, et al. Effect of skin testing and segregation on the prevalence of bovine tuberculosis, and molecular typing of Mycobacterium bovis, in Ethiopia. Vet Rec. 2007;161(23):782-6.

87. Ameni G, Tafess K, Zewde A, Eguale T, Tilahun M, Hailu T, et al. Vaccination of calves with Mycobacterium bovis Bacillus Calmette-Guerin reduces the frequency and severity of lesions of bovine tuberculosis under a natural transmission setting in Ethiopia. Transboundary Emer Dis. 2018;65(1):96-104.

54. Ameni G, Vordermeier M, Aseffa A, Young DB, Hewinson RG. Field evaluation of the efficacy of Mycobacterium bovis bacillus Calmette-Guérin against bovine tuberculosis in neonatal calves in Ethiopia. Clin Vaccine Immunol. 2010;17(10):1533-8.

101. Anderson EC, Doughty WJ, Anderson J. The effect of repeated vaccination in an enzootic foot-and-mouth disease area on the incidence of virus carrier cattle. J Hyg. 1974;73(2):229-35.

52. Awa DN, Njoya A, Tama ACN. Economics of prophylaxis against peste des petits ruminants and gastrointestinal helminthosis in small ruminants in North Cameroon. Trop Anim Health Prod. 2000;32(6):391-403.

67. Ayele B, Tigre W, Deressa B. Epidemiology and financial loss estimation of blackleg on smallholder cattle herders in Kembata Tambaro zone, Southern Ethiopia. Springerplus. 2016;5(1):1822.

65. Ayelet G, Abate Y, Sisay T, Nigussie H, Gelaye E, Jemberie S, et al. Lumpy skin disease: preliminary vaccine efficacy assessment and overview on outbreak impact in dairy cattle at Debre Zeit, central Ethiopia. Antiviral Research. 2013;98(2):261-5.

66. Ayelet G, Haftu R, Jemberie S, Belay A, Gelaye E, Sibhat B, et al. Lumpy skin disease in cattle in central Ethiopia: outbreak investigation and isolation and molecular detection of the virus. Revue Scientifique et Technique. 2014;33(3):877-87.

34. Ba SB, Udo HMJ, Zwart D. Impact of veterinary treatments on goat mortality and offtake in the semi-arid area of Mali. Small Ruminant Res. 1996;19(1):1-8.

74. Barasa M, Catley A, Machuchu D, Laqua H, Puot E, Tap Kot D, et al. Foot-and-mouth disease vaccination in South Sudan: benefit-cost analysis and livelihoods impact. Transboundary Emer Dis. 2008;55(8):339-51.

41. Berggren SA. Field experiment with BCG vaccine in Malawi. BR VET J. 1981;137(1):88-94.

42. Berggren SA. Incidence of Tuberculosis in BCG Vaccinated and Control Cattle in Relation to Age Distribution in Malawi. BR VET J. 1977;133(5):490-4.

62. Bertram MR, Delgado A, Pauszek SJ, Smoliga GR, Brito B, Stenfeldt C, et al. Effect of vaccination on cattle subclinically infected with foot-and-mouth disease virus in Cameroon. Prev Vet Med. 2018;155:1-10.

36. Camus E. [Brucellosis vaccination in cows of the northern Ivory Coast : technic and results]. REV ELEV MED VET PAYS TROP. 1980;33(4):363-9.

86. Catley A, Abebe D, Admassu B, Bekele G, Abera B, Eshete G, et al. Impact of drought-related vaccination on livestock mortality in pastoralist areas of Ethiopia. Disasters. 2009;33(4):665-85.

102. Caufour P, Rufael T, Lamien CE, Lancelot R, Kidane M, Awel D, et al. Protective efficacy of a single immunization with capripoxvirus-vectored recombinant peste des petits ruminants vaccines in presence of pre-existing immunity. Vaccine. 2014;32(30):3772-9.

46. Chema S. Vaccination as a method of foot and mouth disease control: an appraisal of the success achieved in Kenya, 1968-1973. BULL OFF INT EPIZOOT. 1975;83(3-4):195-209.

110. Cloete M, Dungu B, Van Staden LI, Ismail-Cassim N, Vosloo W. Evaluation of different adjuvants for foot-and-mouth disease vaccine containing all the SAT serotypes. Onderstepoort J Vet Res. 2008;75(1):17-31.

38. Coackley W, Weston SJ. Studies on Clostridium chauvoei vaccine in Kenya. J Comp Pathol. 1957;67(2):157-64.

89. Domenech J. [Contagious bovine pleuropneumonia in Ethiopia. Epidemiology and vaccination]. REV ELEV MED VET PAYS TROP. 1979;32(2):143-7.

44. Doutre MP, Chambron J. [Value of the immunity produced by an antiperipneumonic freeze-dried vaccine prepared with the T1 strain]. REV ELEV MED VET PAYS TROP. 1970;23(2):163-79.

45. Doutre MP, Chambron J, Bourdin P. [Value of the immunity produced by a mixed antirinderpest-antiperipneumonia vaccine freeze dried vaccine prepared with the T1 (S-R) strain]. REV ELEV MED VET PAYS TROP. 1972;25(1):1-14.

83. ElArbi AS, Kane Y, Metras R, Hammami P, Ciss M, Beye A, et al. PPR Control in a Sahelian Setting: What Vaccination Strategy for Mauritania? Front Vet Sci. 2019;6:242.

40. Ellwood DC, Waddington FG. A Second Experiment to Challenge the Resistance to Tuberculosis in B.C.G. Vaccinated Cattle in Malawi. BR VET J. 1972;128(12):619-26.

37. Emslie FR, Nel JR. An overview of the eradication of Brucella melitensis from KwaZulu-Natal. Onderstepoort J Vet Res. 2002;69(2):123-7.

68. Fadiga M, Jost C, Ihedioha J. Financial costs of disease burden, morbidity and mortality from priority livestock diseases in Nigeria: Disease burden and cost-benefit analysis of targeted interventions. Nairobi, Kenya: ILRI; 2013. Contract No.: ILRI Research Report 33.

79. Garba SA, Terry RJ, Adegboye DS, Lamorde AG, Abalaka JA. The choice of adjuvants in Mycoplasma vaccines. Microbios. 1989;57(230):15-9.

104. Gari G, Abie G, Gizaw D, Wubete A, Kidane M, Asgedom H, et al. Evaluation of the safety, immunogenicity and efficacy of three capripoxvirus vaccine strains against lumpy skin disease virus. Vaccine. 2015;33(28):3256-61.

75. Gari G, Bonnet P, Roger F, Waret-Szkuta A. Epidemiological aspects and financial impact of lumpy skin disease in Ethiopia. Prev Vet Med. 2011;102(4):274-83.

93. Gilbert FR, Windsor RS. The immunizing dose of T1 strain Mycoplasma mycoides against contagious bovine pleuropneumonia. Trop Anim Health Prod. 1971;3:71-6.

92. Gilbert FR, Davies G, Read WC, Turner GR. The efficacy of T1 strain broth vaccine against contagious bovine pleuropneumonia: in-contact trials carried out six and twelve months after primary vaccination. Veterinary Record. 1970;86:29-33.

94. Gray MA, Simam P, Smith GR. Observations on experimental inactivated vaccines for contagious bovine pleuropneumonia. J Hyg. 1986;97(2):305-15.

48. Gummow B, Mapham PH. A stochastic partial-budget analysis of an experimental Pasteurella haemolytica feedlot vaccine trial. Prev Vet Med. 2000;43(1):29-42.

105. Holzer B, Taylor G, Rajko-Nenow P, Hodgson S, Okoth E, Herbert R, et al. Determination of the minimum fully protective dose of adenovirus-based DIVA vaccine against peste des petits ruminants virus challenge in East African goats. Veterinary Research. 2016;47:20.

109. Hübschle OJB, Tjipura-Zaire G, Abusugra I, Di Francesca G, Mettler F, Pini A, et al. Experimental field trial with an immunostimulating complex (ISCOM) vaccine against contagious bovine pleuropneumonia. Journal of Veterinary Medicine, Series B. 2003;50(6):298-303.

108. Huebschle OJB, Ayling RD, Godinho K, Lukhele O, Tjipura-Zaire G, Rowan TG, et al. Danofloxacin (Advocin™) reduces the spread of contagious bovine pleuropneumonia to healthy in-contact cattle. Res Vet Sci. 2006;81(3):304-9.

80. Jarikre TA, Taiwo JO, Emikpe BO, Akpavie SO. Protective effect of intranasal peste des petits ruminants virus and bacterin vaccinations: Clinical, hematological, serological, and serum oxidative stress changes in challenged goats. Vet World. 2019;12(7):945-50.

60. Jemberu WT, Molla W, Fentie T. A randomized controlled field trial assessing foot and mouth disease vaccine effectiveness in Gondar Zuria district, Northwest Ethiopia. Prev Vet Med. 2020;183:105136.

107. Jemberu WT, Mourits M, Rushton J, Hogeveen H. Cost-benefit analysis of foot and mouth disease control in Ethiopia. Prev Vet Med. 2016;132:67-82.

47. Kairu-Wanyoike SW, Kaitibie S, Heffernan C, Taylor NM, Gitau GK, Kiara H, et al. Willingness to pay for contagious bovine pleuropneumonia vaccination in Narok South District of Kenya. Prev Vet Med. 2014;115(3-4):130-42.

69. Kairu-Wanyoike SW, Taylor NM, Heffernan C, Kiara H. Micro-economic analysis of the potential impact of contagious bovine pleuropneumonia and its control by vaccination in Narok district of Kenya. Livest Sci. 2017;197:61-72.

47. Lancelot R, Lesnoff M, McDermott JJ. Use of Akaike information criteria for model selection and inference. An application to assess prevention of gastrointestinal parasitism and respiratory mortality of Guinean goats in Kolda, Senegal. Prev Vet Med. 2002;55(4):217-40.

113. Lazarus DD, Peta F, Blight D, Van Heerden J, Mutowembwa PB, Heath L, et al. Efficacy of a foot-and-mouth disease vaccine against a heterologous SAT1 virus challenge in goats. Vaccine. 2020;38(24):4006-15.

43. le Roex N, Cooper D, van Helden PD, Hoal EG, Jolles AE. Disease Control in Wildlife: Evaluating a Test and Cull Programme for Bovine Tuberculosis in African Buffalo. Transboundary Emer Dis. 2016;63(6):647-57.

59. Lendzele SS, Mavoungou JF, Burinyuy KA, Armel KA, Dickmu SJ, Young JR, et al. Efficacy and application of a novel topical anaesthetic wound formulation for treating cattle with Foot-and-Mouth disease: A field trial in Cameroon. Transboundary Emer Dis. 2020.

49. Lesnoff M, Lancelot R, Tillard E, Dohoo IR. A steady-state approach of benefit-cost analysis with a periodic leslie-matrix model. Presentation and application to the evaluation of a sheep-diseases preventive scheme in Kolda, Senegal. Prev Vet Med. 2000;46(2):113-28.

99. Lindley EP. Simultaneous vaccination of cattle with contagious bovine pleuropneumonia and goat-adapted rinderpest vaccine. Bulletin of epizootic diseases of Africa. 1967;15(3):221-6.

61. Lyons NA, Stärk KDC, van Maanen C, Thomas SL, Chepkwony EC, Sangula AK, et al. Epidemiological analysis of an outbreak of foot-and-mouth disease (serotype SAT2) on a large dairy farm in Kenya using regular vaccination. Acta Trop. 2015;143:103-11.

111. Maree FF, Nsamba P, Mutowembwa P, Rotherham LS, Esterhuysen J, Scott K. Intra-serotype SAT2 chimeric foot-and-mouth disease vaccine protects cattle against FMDV challenge. Vaccine. 2015;33(25):2909-16.

98. Mariner JC, McDermott J, Heesterbeek JAP, Thomson G, Roeder PL, Martin SW. A heterogeneous population model for contagious bovine pleuropneumonia transmission and control in pastoral communities of East Africa. Prev Vet Med. 2006;73(1):75-91.

97. Mariner JC, McDermott J, Heesterbeek JA, Thomson G, Martin SW. A model of contagious bovine pleuropneumonia transmission dynamics in East Africa. Prev Vet Med. 2006;73(1):55-74.

84. Martrenchar A, Zoyem N, Diallo A. Experimental study of a mixed vaccine against peste des petits ruminants and capripox infection in goats in northern Cameroon. Small Ruminant Res. 1997;26:39-44.

51. Martrenchar A, Zoyem N, Njoya A, Tama A-CN, Bouchel D, Diallo A. Field study of a homologous vaccine against peste des petits ruminants in northern Cameroon. Small Ruminant Res. 1999;31:277-80.

95. Masiga WN. Comparative susceptibility of Bos indicus and Bos taurus to contagious bovine pleuropneumonia, and the efficacy of the T1 broth culture vaccine. Vet Rec. 1972;90(18):499-502.

76. Molla W, de Jong MCM, Gari G, Frankena K. Economic impact of lumpy skin disease and cost effectiveness of vaccination for the control of outbreaks in Ethiopia. Prev Vet Med. 2017;147:100-7.

64. Molla W, Frankena K, Gari G, de Jong MCM. Field study on the use of vaccination to control the occurrence of lumpy skin disease in Ethiopian cattle. Prev Vet Med. 2017;147:34-41.

56. Muuka G, Otina B, Wesonga H, Bowa B, Gicheru N, Stuke K, et al. Evaluation of new generation macrolides for the treatment and metaphylaxis of contagious bovine pleuropneumonia (CBPP) in cattle experimentally infected with Mycoplasma mycoides subspecies mycoides. BMC Vet Res. 2019;15(1):451.

114. Mwacalimba KK, Mumba C, Munyeme M. Cost benefit analysis of tuberculosis control in wildlife-livestock interface areas of Southern Zambia. Prev Vet Med. 2013;110(2):274-9.

103. Ngichabe CK, Wamwayi HM, Ndung'u EK, Mirangi PK, Bostock CJ, Black DN, et al. Long term immunity in African cattle vaccinated with a recombinant capripox-rinderpest virus vaccine. Epidemiol Infect. 2002;128(2):343-9.

77. Niang M, Sery A, Doucoure M, Kone M, N'Diaye M, Amanfu W, et al. Experimental studies on the effect of long-acting oxytetracycline treatment in the development of sequestra in contagious bovine pleuropneumonia-infected cattle. Journal of Veterinary Medicine and Animal Health. 2010;2:35-45.

85. Njoya A, Awa DN, Chupamom J. The effects of a strategic supplementation and prophylaxis on the reproductive performance of primiparous Fulbe ewes in the semi-arid zone of Cameroon. Small Ruminant Res. 2005;56(1-3):21-9.

90. Nkando I, Ndinda J, Kuria J, Naessens J, Mbithi F, Schnier C, et al. Efficacy of two vaccine formulations against contagious bovine pleuropneumonia (CBPP) in Kenyan indigenous cattle. Res Vet Sci. 2012;93(2):568-73.

91. Nkando I, Perez-Casal J, Mwirigi M, Prysliak T, Townsend H, Berberov E, et al. Recombinant Mycoplasma mycoides proteins elicit protective immune responses against contagious bovine pleuropneumonia. Vet Immunol Immunopathol. 2016;171:103-14.

63. Nyaguthii DM, Armson B, Kitala PM, Sanz-Bernardo B, Di Nardo A, Lyons NA. Knowledge and risk factors for foot-and-mouth disease among small-scale dairy farmers in an endemic setting. Veterinary Research. 2019;50(1):33.

106. Onono JO, Wieland B, Rushton J. Estimation of impact of contagious bovine pleuropneumonia on pastoralists in Kenya. Prev Vet Med. 2014;115(3-4):122-9.

72. Renault V, Hambe HA, Van Vlaenderen G, Timmermans E, Mohamed AM, Ethgen O, et al. Economic impact of contagious caprine pleuropneumonia and cost–benefit analysis of the vaccination programmes based on a one-year continuous monitoring of flocks in the arid and semi-arid lands of Kenya. Transboundary Emer Dis. 2019;66(6):2523-36.

53. Reynolds L, Francis P. The effect of PPR control and dipping on village goat populations in southwest Nigeria. ILCA Bulletin. 1988;32:22-7.

88. Roug A, Perez A, Mazet JAK, Clifford DL, VanWormer E, Paul G, et al. Comparison of intervention methods for reducing human exposure to Mycobacterium bovis through milk in pastoralist households of Tanzania. Prev Vet Med. 2014;115(3-4):157-65.

100. Rurangirwa FR, McGuire TC, Mbai L, Ndung'u L, Wambugu A. Preliminary field test of lyophilised contagious caprine pleuropneumonia vaccine. Research in Veterinary Science. 1991;50(2):240-1.

112. Scott KA, Rathogwa NM, Capozzo AV, Maree FF. Evaluation of immune responses of stabilised SAT2 antigens of foot-and-mouth disease in cattle. Vaccine. 2017;35(40):5426-33.

81. Souley Kouato B, Thys E, Renault V, Abatih E, Marichatou H, Issa S, et al. Spatio-temporal patterns of foot-and-mouth disease transmission in cattle between 2007 and 2015 and quantitative assessment of the economic impact of the disease in Niger. Transboundary Emer Dis. 2018;65(4):1049-66.

82. Stem C. An economic analysis of the prevention of peste des petits ruminants in Nigerien goats. Prev Vet Med. 1993;16(2):141-50.

57. Suleiman A, Bello M, Dzikwi AA, Talba AM, Grema HA, Geidam YA. Serological prevalence of contagious bovine pleuropneumonia in agro-pastoral areas of Nigeria. Trop Anim Health Prod. 2015;47(6):1033-42.

117. Tambi NE, Maina WO, Ndi C. An estimation of the economic impact of contagious bovine pleuropneumonia in Africa. Revue Scientifique et Technique. 2006;25(3):999-1011.

78. Tambuwal FM, Egwu GO, Shittu A, Sharubutu GH, Umaru MA, Umar HU, et al. Vaccination Coverage and Prevalence of Contagious Bovine Pleuropneumonia (1999 - 2008) in Two Transboundary States of North-Western Nigeria. Nigerian Veterinary Journal. 2011;32(3):169-73.

115. Thiaucourt F, Aboubakar Y, Wesonga H, Manso-Silvan L, Blanchard A. Contagious bovine pleuropneumonia vaccines and control strategies: recent data. Developments in Biologicals. 2004;119:99-111.

116. Thiaucourt F, Yaya A, Wesonga H, Huebschle OJ, Tulasne JJ, Provost A. Contagious bovine pleuropneumonia. A reassessment of the efficacy of vaccines used in Africa. Annals of the New York Academy of Sciences. 2000;916:71-80.

39. Waddington FG, Ellwood DC. An Experiment to Challenge the Resistance to Tuberculosis in B.C.G. Vaccinated Cattle in Malawi. BR VET J. 1972;128(11):541-52.

96. Wesonga HO, Thiaucourt F. Experimental Studies on the Efficacy of T1sr and T1/44 Vaccine Strains of Mycoplasma mycoides Subspecies mycoides (Small Colony) against a Field Isolate Causing Contagious Bovine Pleuropneumonia in Kenya - Effect of a Revaccination. REV ELEV MED VET PAYS TROP. 2000;53(4):313-8.

50. Wosu LO, Okiri JE, Enwezor PA. Optimal time for vaccination against Peste des petits ruminants (PPR) disease in goats in humid tropical zone in southern Nigeria. Arch Roum Pathol Exp Microbiol. 1990;49(3):283-91.

58. Zerbo LH, Dahourou LD, Sidi M, Ouoba LB, Ouandaogo SH, Bazimo G, et al. Seroprevalence and determinants of contagious bovine pleuropneumonia in cattle in Burkina Faso. Trop Anim Health Prod. 2021;53(1).
